# A random forest based biomarker discovery and power analysis framework for diagnostics research

**DOI:** 10.1186/s12920-020-00826-6

**Published:** 2020-11-23

**Authors:** Animesh Acharjee, Joseph Larkman, Yuanwei Xu, Victor Roth Cardoso, Georgios V. Gkoutos

**Affiliations:** 1 College of Medical and Dental Sciences, Institute of Cancer and Genomic Sciences, Centre for Computational Biology, University of Birmingham, Birmingham, B15 2TT UK; 2 Institute of Translational Medicine, University Hospitals Birmingham NHS, Foundation Trust, Birmingham, B15 2TT UK; 3 NIHR Surgical Reconstruction and Microbiology Research Centre, University Hospital Birmingham, Birmingham, B15 2WB UK; 4 MRC Health Data Research UK (HDR UK), London, UK; 5 NIHR Experimental Cancer Medicine Centre, Birmingham, B15 2TT UK; 6 NIHR Biomedical Research Centre, University Hospital Birmingham, Birmingham, B15 2TT UK

**Keywords:** Random forest, Feature selection, Power study, Biomarker

## Abstract

**Background:**

Biomarker identification is one of the major and important goal of functional genomics and translational medicine studies. Large scale –omics data are increasingly being accumulated and can provide vital means for the identification of biomarkers for the early diagnosis of complex disease and/or for advanced patient/diseases stratification. These tasks are clearly interlinked, and it is essential that an unbiased and stable methodology is applied in order to address them. Although, recently, many, primarily machine learning based, biomarker identification approaches have been developed, the exploration of potential associations between biomarker identification and the design of future experiments remains a challenge.

**Methods:**

In this study, using both simulated and published experimentally derived datasets, we assessed the performance of several state-of-the-art Random Forest (RF) based decision approaches, namely the Boruta method, the permutation based feature selection without correction method, the permutation based feature selection with correction method, and the backward elimination based feature selection method. Moreover, we conducted a power analysis to estimate the number of samples required for potential future studies.

**Results:**

We present a number of different RF based stable feature selection methods and compare their performances using simulated, as well as published, experimentally derived, datasets. Across all of the scenarios considered, we found the Boruta method to be the most stable methodology, whilst the Permutation (Raw) approach offered the largest number of relevant features, when allowed to stabilise over a number of iterations. Finally, we developed and made available a web interface (https://joelarkman.shinyapps.io/PowerTools/) to streamline power calculations thereby aiding the design of potential future studies within a translational medicine context.

**Conclusions:**

We developed a RF-based biomarker discovery framework and provide a web interface for our framework, termed PowerTools, that caters the design of appropriate and cost-effective subsequent future omics study.

## Background

Over the last few years there has been lots of emphasis on the high dimensional omics data generation, including untargeted –omics datasets, like transcriptomics [1, 2] metabolomics [3, 4], proteomics [5, 6], microbiomes [7–9], as well as deep phenotyping [10]. As a consequence, large amount of data is routinely being accumulated, which needs to be integrated and analysed so as to facilitate the identification of relevant markers. If the biomarkers identified from the different-omics datasets are robust, reproducible and indicative, then they can be potentially useful for patient/disease stratification [11, 12] and can also provide powerful clinical relevant insights as diagnostic or prognostic tools. Selecting relevant markers or features from high dimensional datasets is defined as feature or variable selection [13] and requires a robust statistical or computational workflow [14].

The application of machine learning methods for feature selection is a well-established approach [14]. Lately, decision tree based statistical machine learning methods, for example, Random Forest (RF) [15], have gained prominence. RF is an ensemble learning method, which has been applied successfully on multiple high dimensional omics studies including transcriptomics [16], metabolomics [17], methylation [18] and proteomics [19]. The objective of these studies has been either the prediction or the selection of important features that can serve as potential biomarkers and that can be potentially employed for patient stratification. The random forest algorithm is a powerful prediction method that is known to be able to capture complex dependency patterns between the outcome and the covariates. The latter feature renders random forest a promising candidate for developing prediction methods tailored to the challenges multi-omics data analysis entails.

In the case of omics analysis, it is important to employ feature selection procedures that are systematic and data driven so to avoid any bias selection. RF, coupled with other feature selection methods, has successfully been applied for such tasks, for example, in studies related to the selection of genes and metabolites [20] and the selection of lipids and metabolites [21]. More recently, Degenhardt et al. [26] performed a simulation study as well as applied a number of different RF-based method on publicly available experimental datasets. However, their results, due to the lack of an assessment and validation of the effect/weight of the identified markers, have limited value within the context of a ‘study design’ or a ‘power analysis’ for potential future translation research studies. Other studies have focused on these aspects using metabolomics [22, 23] and transcriptomics datasets [24], however, these approaches are tailored made to specific -omics types and often fail to properly relate, using stable feature selection procedures, to power calculations for identified putative biomarkers.

In this study, we have performed an extensive simulation using RF based feature selection methods, namely the Boruta [25], the permutation based feature selection [26], the permutation based feature selection with correction [26], and the backward elimination based feature selection [27] methods, both in a regression and classification context, so as to assess their feature selection and prediction error abilities. We further assessed the performance of these methods over experimentally derived datasets in an effort to understand their performance over disparate -omics dataset paradigms. We also developed a workflow to identify the number of samples required for a future study using the stable biomarkers that were identified in the first task. Finally, we developed a web interface, termed PowerTools, to streamline power calculations, encompassed by our approach, offering the potential for designing appropriate and cost-effective subsequent future omics study designs.

## Methods

### Random forest (RF)

Random Forest (RF) [15] is an ensemble-based machine learning (ML) method, based on a decision tree algorithm, that can be used for both classification as well as regression based analysis. Typically, around two-thirds of a particular study samples are used for the model fitting or training while the remaining one-third is used for model testing, termed as the out-of-bag (OOB) samples. OOB is used to quantify the model performance. For the case of classification, the prediction performance can be quantified in terms of the rate at which OOB samples are misclassified across multiple classes or the OOB error. For the case of regression, the average distance between OOB predictions and the true continuous response variable can be quantified using the mean squared error (MSE) metric. The contribution from each variable to the final model is quantified as a ranked measure of variable importance (detailed information is provided in the methods sections in Additional file 1).

### RF feature selection methods

Four methods, namely, the Boruta [25], the permutation based feature selection [26], the permutation based feature selection with correction [26], and the backward elimination based feature selection [27] methods, were applied so as to automatically select important features from the aforementioned ranked list RF generates. The details of the statistical basis of each of these approaches is outlined in the following section.

### Boruta

Boruta [25] compares the feature importance values estimated for the real predictor variables, against the variables generated by the permutation of these variables across observations. Variables, generated by permutation, are termed “shadow” variables. For each run, a RF is trained using a double length set of predictor variables comprised of an equal number of true and shadow variables. For each of the real predictor variables, a statistical test is conducted comparing its importance in relation to the maximum importance value achieved by a shadow variable. Variables with significantly larger or smaller importance values are defined by the algorithm as important or unimportant, respectively. In subsequent runs, all unimportant and shadow variables are removed, and the process is repeated until all variables have been classified or a specified maximum number of runs is reached.

### Permutation based feature selection

Permutation testing is an established approach for approximating a significance level or threshold for the selection of a subset of associated features derived from a RF model [21, 26]. While RF quantifies the importance of the features that explain the variation present in the outcome variable (either quantitative or binary), it fails to provide a significance label for the selected features. The aim of including permutation within the RF model was to automatically select features based on the significance label estimated from the input data. We permutated the outcome variable (either quantitative or binary) separately and applied a RF model for each case. The RF model was applied 1000 times over 1000 different outcome variable randomizations and for each analysis we estimated the variance or class error explained by the RF model and compared with the true distribution. [21, 45, 21] provides the details of the methodology adopted.

Finally, permutation testing was implemented using both raw (uncorrected) p-values, as well corrected ones, for multiple hypothesis testing using the Benjamini–Hochberg procedure (corrected) [28]. For both implementations, a threshold p-value of 0.05 was used to determine statistical significance.

### Recursive feature elimination

Recursive feature elimination (RFE) [27] forms an approach aiming to determine the smallest subset of variables that produce an effective model with a good prediction accuracy. The methodology it adopts involves the iterative fitting of RF, where upon each iteration, a specified proportion of variables, with the smallest variable importance, is discarded. This process is applied recursively until only a single variable remains available as input. At each iteration, the model performance is assessed in terms of the out-of-bag error, when RF is used in a classification capacity, or mean squared error (MSE) for regression forests. The set of variables, leading to the generation of the smallest, or one within a specified minimum range, RF error, are ultimately selected. In this study, we used the R package varSelRF [27] for the implementation of this method. Moreover, we modified the constituent functions of varSelRF to accept a continuous y variable input and used MSE for the model assessment so as to facilitate the feature selection when RF is used in a regression capacity.

## Statistical analysis

### Module 1: stable feature selection

**Fig. 1:**
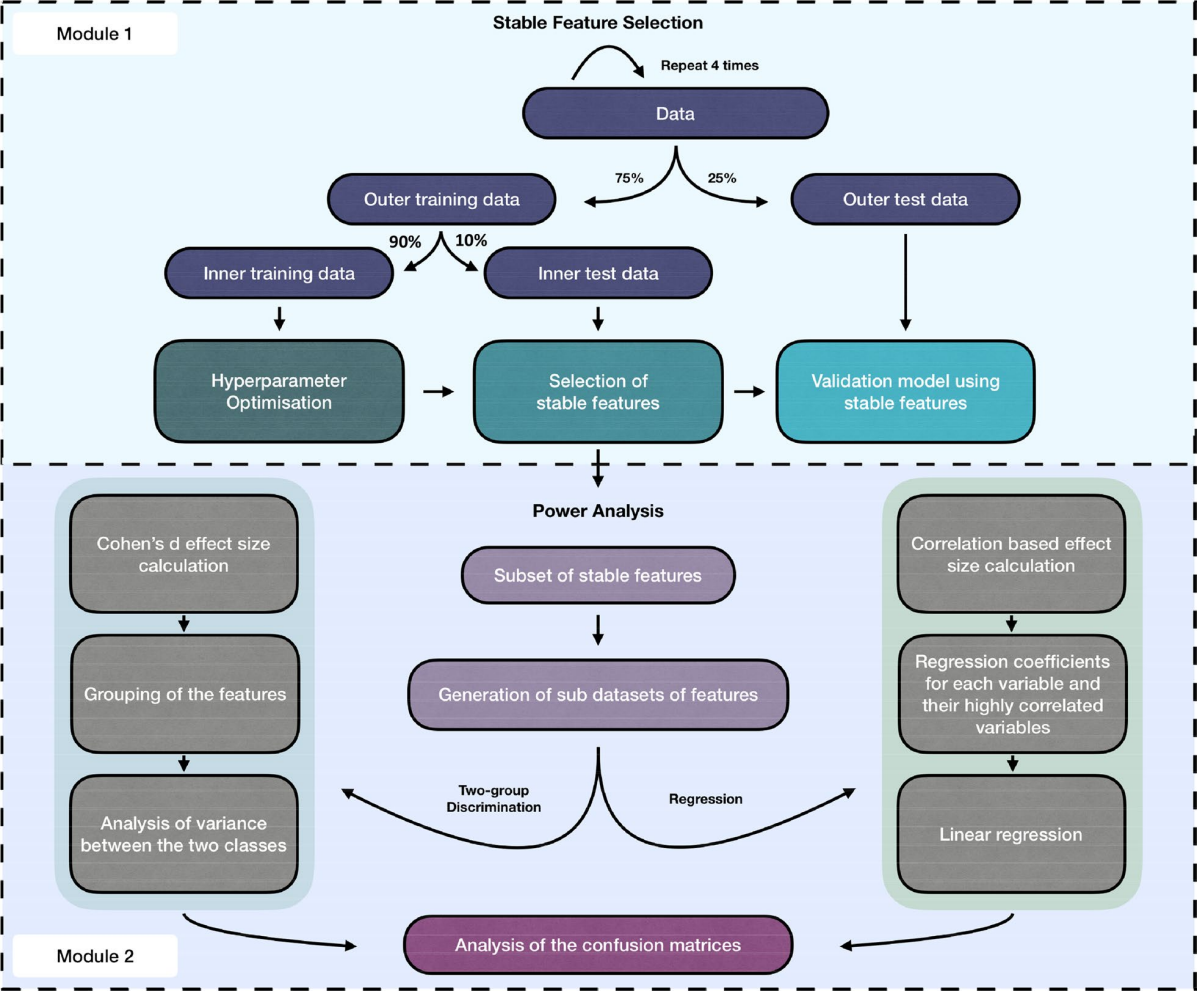
Schematic diagram of the simulation set up and the published experimentally derived (real) data analysis

We divided our analysis approach into two modules. Module one incorporates a nested cross validation (CV) procedure [29–32], which is summarised in Fig. 1 (Module 1)**.** First, a training:testing ratio of 75:25 is used to generate the outer train and test data subsets. The whole process is repeated 100 times with multiple repeating folds. The outer train subset is then subject to a further tenfold CV, where one-tenth of the data (inner train) is used for the hyper parameter optimisation (additional information is provided in the methods section in the Additional file 1). The remaining nine-tenth of the data, forming the inner test subset, is then used for the application of 100 iterations of feature selection. For each iteration, a RF is trained and each of the aforementioned methods is applied to identify a subset of important features. Stable features are then defined as those identified by a particular method following a number of iterations greater than a specified stringency value. In the present study, features, selected in > 5/100 iterations, are determined low stringency (LS) stable features and ones, selected following a minimum of 90/100 iterations, are considered high stringency (HS) stable features. The entire procedure is repeated four times, each time using a modified outer loop split, such that each sample appears once within the outer test data subset. The values of each feature, selected by each method, specifying the predictive power, the prediction error, and the frequency, are then averaged across the four outer loop repeats. The full nested CV procedure was applied to simulated data as well as three published experimentally derived datasets. Due to sample size limitations, CV was not conducted for one of the selected experimentally derived datasets.

### Module 2: power analysis

We implemented a flexible approach to facilitate the power analysis and to determine the sample size, based on the functions designed and described by Blaise et al., [23]. We included both datasets with a continuous outcome variable (regression) as well as ones with a two-group (binary) classification outcome. Furthermore, the correlation structure of the input data was explicitly modelled, in order to capture any multicollinearity between variables. An overview of the approach is represented schematically in Fig. 1 (Module 2) and described briefly in the following section. (detailed information is provided in the methods section in the Additional file 1).

The synthetic data was first generated using a multivariate log-normal distribution, namely the R library MASS ‘mvrnorm’ function. From the resulted simulated datasets, a subset of the data was considered containing a specified series of sample sizes (for example: 5, 10, 15, 50, 100 etc.). In the case of regression, for each of the variables assessed, a continuous outcome is generated that relates the assessed variable with a Pearson correlation value equal to that of its relation to the real outcome (effect size). The simulated outcome variable is then regressed against each variable to produce a set of p-values describing each variable’s association with the outcome.

For the two-group (binary) classification case, two datasets are produced for each specified sample size (one for each group) and a specified Cohens d effect size [33] is introduced to one of them and its highly correlated partners. A one-way ANOVA is then conducted for each variable, comparing the intra and inter group variances, and producing a set of p-values describing the variables with statistically significant variances.

In either case, true/false positive metrics are then determined by comparing the set of statistically significant variables to a set containing the variable chosen for analysis and its highly correlated partners.

Furthermore, the R implementation of the PowerTools builds upon the functionality of the original functions in a bifold manner. First, each variable is automatically assessed using its true effect size; in the case of regression, the true effect size of a variable is estimated according to its correlation with the true outcome variable, whilst for a two-group classification, the observed Cohen’s d effect size is computed. Second, highly correlated variables can be optionally grouped together and only the member of each group, with the largest effect size, is used as a representative group member thereby facilitating the identification of a smaller subset of potential biomarkers.

### Dataset description

We used simulated data, as well as published experimentally derived datasets. Table 1 provides a detailed description of these datasets. The data simulation was performed based on the uniform as well as the normal distribution.

**Table 1:** A list of the published datasets used in this study

RF mode	Dataset type	Sample number (N)	Feature number (p)	Outcome variable	Pubmed ID	References
*Regression*	*Metabolomics*	*73*	*196*	*Relative liver weight*	*28,185,575*	[21]
	*Lipidomics*	*40*	*219*	*infant milk amount*	*28,190,990*	[35]
*Classification*	*Metabolomics*	*73*	*196*	*Relative liver weight class (below or above the mean value)*	*28,185,575*	[21]
	*Transcriptomics*	*68*	*414*	*Colorectal cancer (CRC) stages*	*27,176,004*	[36]
	*Transcriptomics*	*20*	*1386*	*Primary Sclerosing Cholangitis (PSC) vs. Ulcerative colitis (UC)*	*32,016,358*	[37]
	*Transcriptomics*	*40*	*25,697*	*OB/OB vs. wild type genotype mouse*	*32,646,215*	[38]

For each of the RF models, two datasets was considered and the model outcome was compared with the published results

#### Uniform distribution-based data simulation

Simulation data, featuring correlated predictor variables and a quantitative outcome variable, were generated using a nonlinear regression model, according to previously reported methods [26, 34]. Specifically, the simulation strategy and its associated equations, outlined by Degenhardt et al., [26] , was adapted and implemented, with minor parameter modifications. A single simulation scheme was incorporated and sixty (six groups of ten) correlated variables were generated, alongside additional uncorrelated variables, to produce a dataset of 5000 predictor variables.

First, six uniformly distributed variables, ${x}_{1}, {x}_{2}, {x}_{3}, {x}_{4}, {x}_{5}$ and ${x}_{6}$, were sampled individually from $U(\mathrm{0,1})$. The correlated predictor variables were then generated according to the equation:

1 $$V_{i}^{\left( j \right)} = x_{i} + \left( {0.01 + \frac{{0.5\left( {j - 1} \right)}}{n - 1}} \right) \cdot N\left( {0,0.3} \right)$$

for $j=1,...,10$ and $i=1,...,6$, where ${V}_{i}^{(j)}$ denotes the $jth$ variable in group $i$ and the correlation between ${V}_{i}^{(j)}$ and $x$ decreases as $j$ increases. Conversely, values for the uncorrelated predictor variables were simply sampled from the uniform distribution $U(\mathrm{0,1})$.

The variables ${x}_{1}, {x}_{2}$ and ${x}_{3}$ were also used to generate the quantitative outcome variable $y$ according to the equation:

2 $$y = 0.25exp \left( {4x_{1} } \right) + \frac{4}{{1 + exp\left( { - 20\left( {x_{2} - 0.5} \right)} \right)}} + 3x_{3} + N\left( {0,0.2} \right)$$

where y correlates decreasingly with variables ${x}_{1}, {x}_{2}$ and ${x}_{3}.$

The simulation scheme, outlined above, was repeated iteratively so as to produce a final simulation dataset consisting 200 observations, 5000 predictor variables and a quantitative outcome variable correlated with only the first three groups of correlated predictor variables. Additionally, the quantitative outcome variable was adapted for a binary classification context, whereby observations with a ‘y’ value, below the mean of the outcome variable set, was assigned to group one, while observations, associated with a ‘y’ value above the mean, were assigned to group two. Consequently, the same simulation protocol was used to facilitate the assessment of feature selection and power analysis in both a classification and regression context.

#### Normal distribution-based data simulation

Six variables x_i(i = 1…6) were sampled from the standard normal distribution and the outcome variable ‘y’ was calculated according to Eq. 2. The median was used as a cut-off value for the class labels so as to obtain a more balanced assignment of classes.

### Publicly available datasets

A summary of the published, experimentally derived datasets is presented in Table 1.

### Software and code availability

We used the R (https://www.r-project.org) v3.5.0 software for statistical computing. Different packages were used for the RF methods, listed in Table 2. The web interface was developed using the R shiny app (http://shiny.rstudio.com/) and is available at: https://joelarkman.shinyapps.io/PowerTools/. All other scripts are have been made freely available in our github repository: https://github.com/joelarkman/RF-FeatureSelection-PowerAnalysis.

**Table 2:** List of the methods and R packages used

Method	R package used	References
Random Forest	randomForest	[15]
Random Forest (Optimised for memory)	ranger	[39]
Boruta	Boruta	[25]
Hyperparameter selection	Caret	[40]
Permutation based feature selection	pomona	[26]
Recursive feature elimination (RFE)	vaSelRF	[27]

## Results

### Regression mode

Data simulations were performed using two different types of distribution, namely a uniform and a random normal distribution.

### Simulation data

**Fig. 2:**
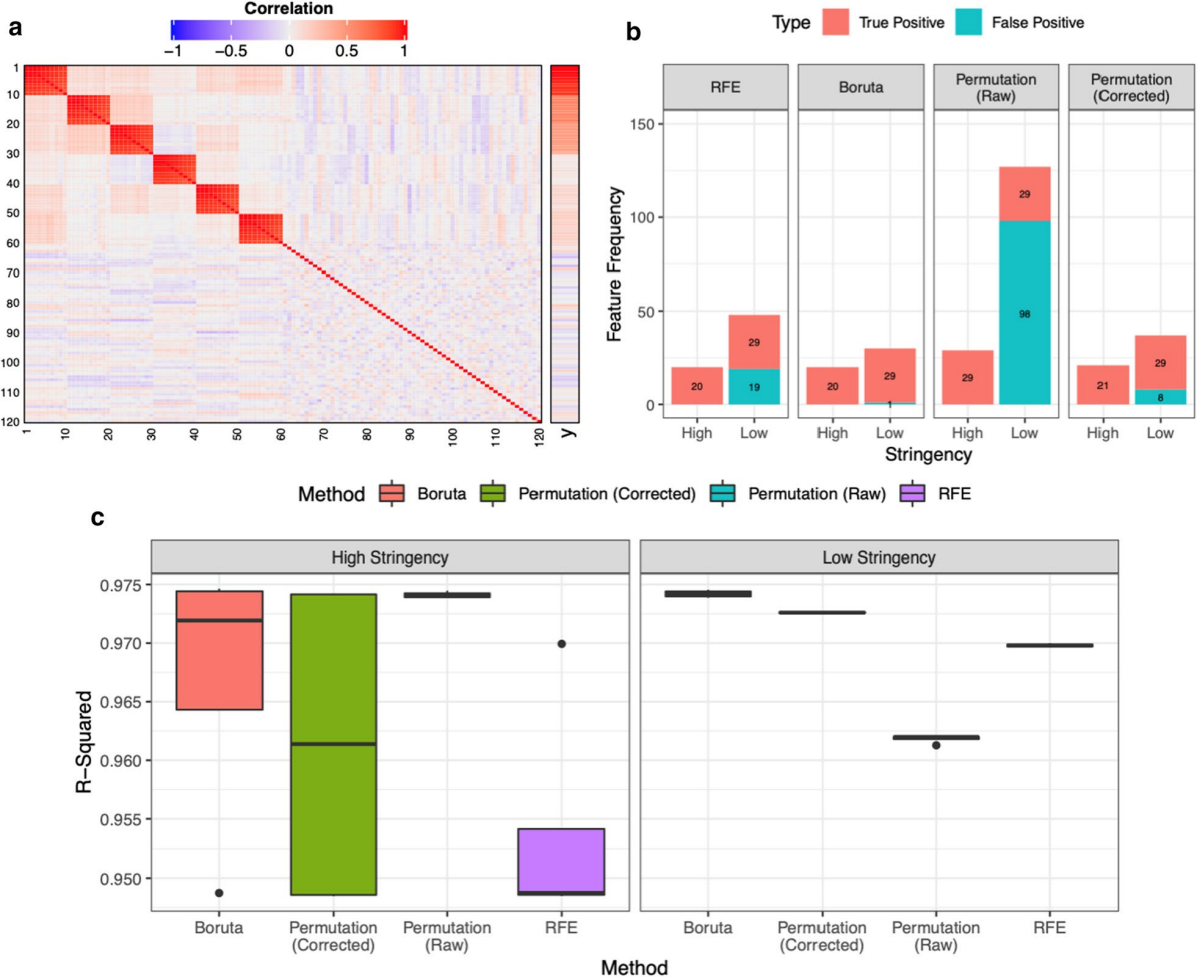
Results from the simulation study in RF regression mode. **a** The structure of the simulated predictor data from uniform distribution and the association with outcome variable (y) is described. Only V1-V120 are shown of full dataset featuring 5000 variables. **b** The number of features stably selected by each approach in at least 5/100 iterations (Low Stringency) or a minimum of 90/100 iterations (High Stringency) are shown. True positive: V1–V30, False positive: V3–V5000. Values describing the number of times each feature is chosen by a particular approach are averaged across those achieved after 100 iterations for each of the four inner loop test datasets. **c** The variance in predictive accuracy (R-Squared), across all four outer loop cross-validation repeats, is shown for RFs trained using only the high or LS stable features selected by each feature selection approach using the relevant inner loop test dataset

We performed a regression analysis considering 5000 predictor variables and a continuous response (y). Figure 2a illustrates a correlation plot of the first 120 features’ relationships, depicting the intra-group correlation for each of the predictor variables as well as the correlation between each feature and outcome variable (y). The first sixty variables formed six highly correlated clusters, whilst the remaining features showed no pattern of correlation between variables. The first three correlated groups exhibited a clear correlation with the outcome variable that decreased in intensity both within and between correlated groups, from V1–V30. All other predictor variables exhibited a negligible correlation with the continuous outcome variable.

The results of RF in regression mode over simulated data are summarised in Fig. 2b, where the feature selection is considered in terms of both HS and LS for stability. In the HS environment, both the Boruta and the RFE methods identified V1–V20, the Permutation (Corrected) method identified V1–V21 and finally the Permutation (Raw) method identified V1–V29. In the LS environment, 29 true positive features (V1–V29) were identified by the Boruta, the RFE and the Permutation (Corrected) methods.

Following the stable feature identification, selected by each method, the performance of the validation models, trained using only these features and held out data, were quantified in terms of predictive accuracy (R-squared). The results of this analysis are shown in Fig. 2c, depicting the predictive performance varying negligibly between the models.

We conducted a power analysis using a subset of the simulation data containing only the six groups of correlated features (V1–V60) and a single non-correlated variable (V4500). The results from this analysis are presented in the Additional file 1, SF2(A), where the groups, containing the 20 Boruta HS stable features are indicated with an asterisk. The six correlated feature groups were successfully detected by the function and grouped together. Each of the group members, with the largest effect size, was then assessed for power, across a broad range of sample sizes, using the continuous outcome variable scheme outlined in Module 2 We compared the features, representing the three groups used to generate y (V1–30), in terms of their effect and sample sizes necessary to achieve maximal power. V1, chosen to represent the first correlated group (V1–V10), had an effect size of 0.82, and achieved a power = 1 with a sample size of ~ 60; the second y, related the second group (V11–V20), was represented by V19 and had an effect size of 0.49 achieving a power = 1 with ~ 140 samples; whilst the third group (V21–30), represented by V21, had an effect size of 0.38 and achieved a power = 1 with a sample size of ~ 225.

The assessment of the power calculations for the non-y-related features revealed similarly negligible effect sizes for V36 (representing the correlated group 3) and V4500. Consequently, the power values calculated for these variables retained a close to zero value across the full range of the considered sample sizes. Contrastingly, V50 and V52 (representing the correlated groups 5 and 6, respectively) each produced an effect size 0.14. Consequently, significant power values could be obtained for these variables at sufficiently large sample sizes. We determined that a sample size of ~ 1990 is necessary to observe power = 1.

The normal distribution results are detailed in the Additional file 1 (SF12). In summary, out of 40 iterations, no features, with a frequency greater than 24, were selected by RFE. In contrast, the Boruta method identified the features V1-V10 with a frequency of at least 30 (Additional file 1, SF12). The R^2^ (% variation explained), using the common stable features, was 98.22%. A list of the R^2^ values across the other methods and data sets is provided with the Additional file 1 (Table 1).

### Dataset 1: metabolomics

A similar approach was applied on a publicly available –omics dataset [21] that features lipid metabolites (Positive DI-MS Lipids) and employs a relative liver weight as a continuous outcome variable (y). Acharjee et al.,[21] identified six metabolites of interest which we considered as known in this study.

**Fig. 3:**
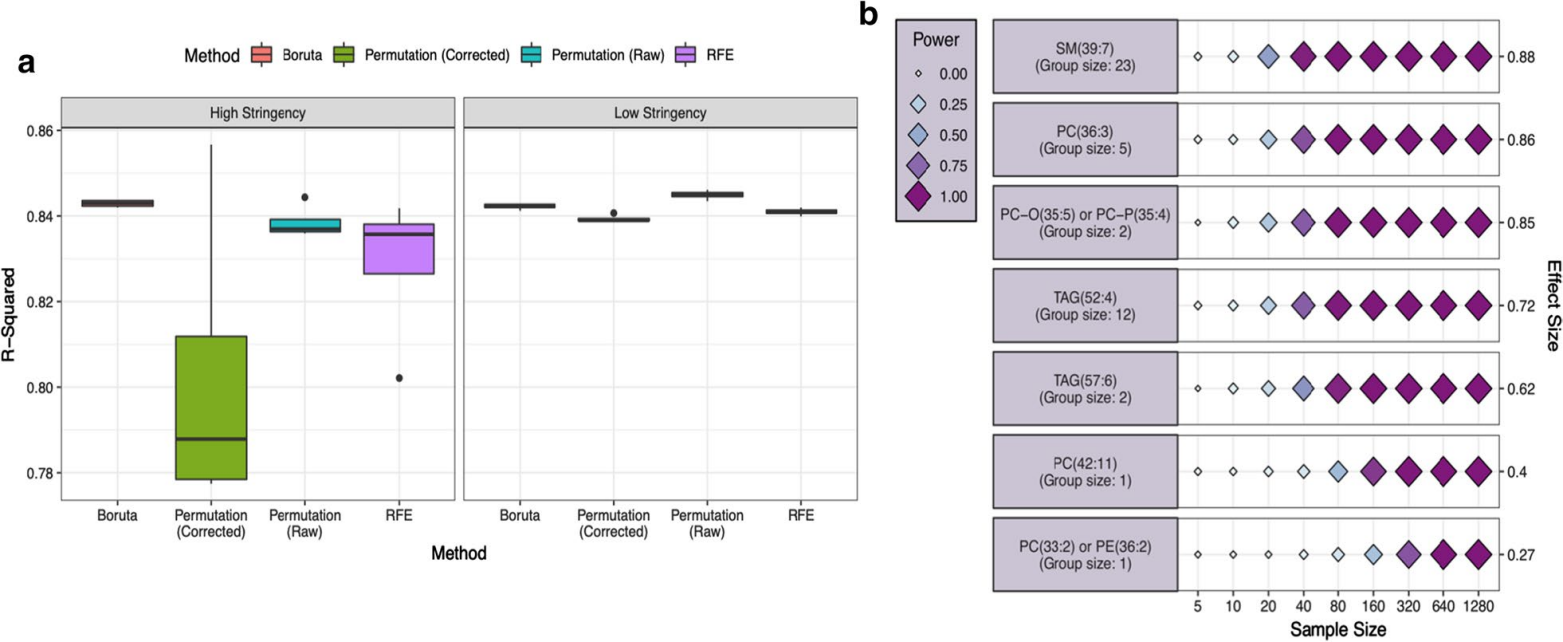
Validation model performance and power analysis of published experimentally derived data 1, regression mode. **a** Boxplots displaying the variance in the observed R-squared value of validation models trained using the stable features selected by each feature selection approach, across four outer-loop CV repeats. Values are shown for models trained using either the features selected by each approach in at least 5/100 iterations (Low Stringency) or a minimum of 90/100 iterations (High Stringency). **b** The three groups of correlated features identified by the power function are represented by the group member with the largest observed effect size. The effect size of each assessed variable is shown along the y axis and a series of sample sizes along the x axis. Power values determined for each effect/sample size combination using a simulated dataset with the same correlation structure as input data and displayed using variably sized/coloured rhombi

The application of the Boruta method resulted in the selection of the largest number of HS features, including three previously known features of interest (Table 3 and Additional file 1, SF3A). In the LS environment, using the Boruta method, we were able to select 40 additional features. In addition, the HS Boruta model achieved the highest R-squared value, slightly exceeding the value it achieved under LS (Fig. 3a). The Permutation (Raw) method resulted in a strong stability across iterations (Table 3), exhibiting consistent predictive performance (R-squared) across validation models (Fig. 3a), and allowing for the identification of two known metabolites under HS. The application of both the RFE and the Permutation (Corrected) methods exhibited poor stability across iterations, retaining only six and three HS features, respectively, despite the methods identifying 94 and 51 features each, under LS (Table 3).

**Table 3:** List of the methods, stringency (high and low) and evaluation criteria used for both regression and classification

RF methods	Stringency	Criteria	Regression			Classification		
			Simulation	Metabolomics	Lipidomics	Simulation	Lipidomics	Transcriptomics-1
RFE	High	TP/Known	20	1	1	10	0	–
		FP/Novel	0	5	0	0	0	0
	Low	TP/Known	29	3	3	29	2	–
		FP/Novel	19	91	8	201	18	14
Boruta	High	TP/Known	20	3	2	11	2	–
		FP/Novel	0	43	6	0	24	19
	Low	TP/Known	29	3	3	29	3	–
		FP/Novel	1	83	34	9	10	39
Permutation (Raw)	High	TP/Known	29	2	2	19	2	–
		FP/Novel	0	24	7	0	10	0
	Low	TP/Known	29	3	3	29	3	–
		FP/Novel	98	68	47	465	35	132
Permutation (Corrected)	High	TP/Known	21	2	1	11	2	–
		FP/Novel	0	1	0	0	6	0
	Low	TP/Known	29	3	3	29	3	–
		FP/Novel	8	48	26	110	46	66

Evaluation criteria are formed by the number of the features identified by each method vs. the known features that are already reported or simulated in the model

**Fig. 4:**
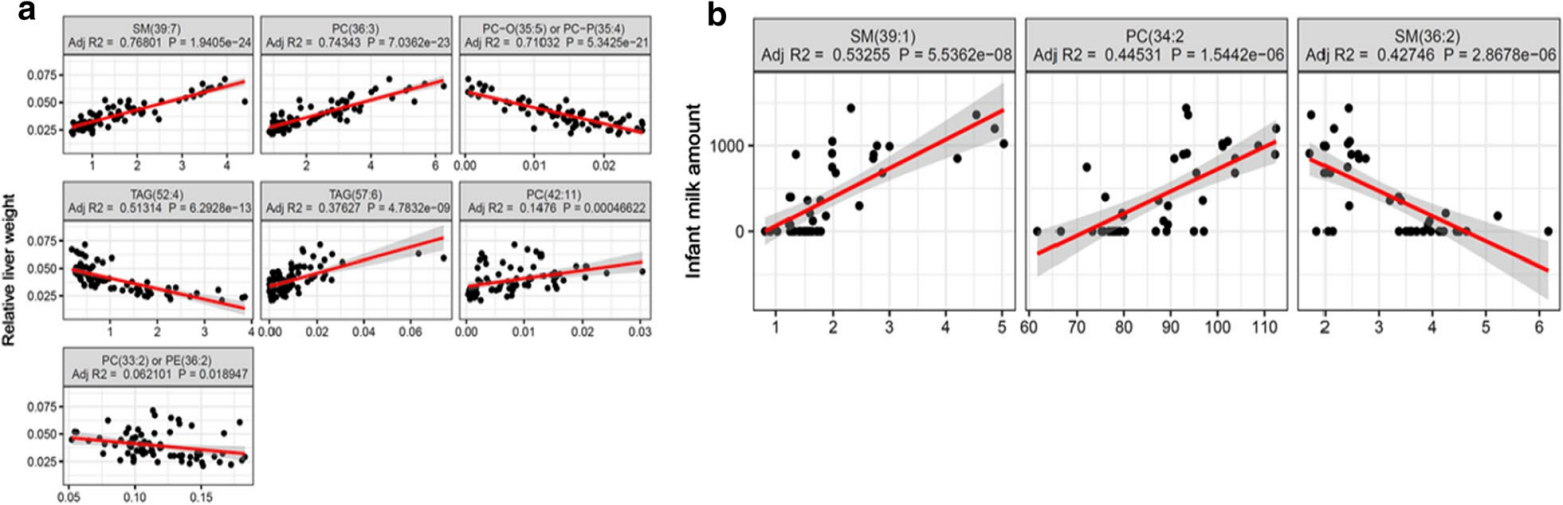
Results from public dataset identified by the module 1 of the workflow is listed above with probability values < 0.05. **a** Stable metabolic markers and their variance explained with relative liver weight is shown. **b** Lipids associated with amount of milk in the 3 m old infants are listed

The Dataset 1 power analysis results are presented in Fig. 3b. PowerTools reduced the 46 HS Boruta features to seven highly correlated groups, and subsequently power calculations were performed for the group member with the largest observed effect size. Five potential biomarkers, with an effect size in excess of 0.6, emerged from this analysis, for which the maximal power values were observed at a sample size between 35 and 45. The two remaining features obtained effect sizes of 0.4 and 0.27 and achieved a maximum power at a sample size of 75 and 620, respectively. The relationship between each of the assessed variables and the continuous outcome variable are displayed in Fig. 4a. All seven variables were deemed statistically significant (P-value < 0.05), whilst adjusted R-squared values ranged from 0.77 for ‘SM(39:7)’ to just 0.06 for ‘PC(33:2) or PE(36:2). The three previously known metabolites identified by the Boruta method, namely ‘PE(42:4)’, ‘PC(40:5)’ and ‘PC(42:9)’, correlated highly with the putative biomarker with the largest effect size ‘SM(39:7)’.

### Dataset 2: lipidomics

The results of the second application of the regression approach, using lipidomic data obtained from 3 months old infants, are summarised in Table 3 and the Additional file 1, SF3B). Previous work identified three lipids, namely PC(35:2), SM(36:2) and SM(39:1) [35], which were considered as known. These metabolites were identified by all of the four feature selection methods, within the LS environment, while the ‘SM(36:2)’ and ‘SM(39:1)’ metabolites were identified by the Boruta and the Permutation (Raw) methods within the HS environment (Additional file 1, SF4A). The RFE and Permutation (Corrected) methods failed to achieve stability over this data, retaining only but a single feature, namely ‘SM(39:1)’, within the HS environment. In total, eight features were selected by the Boruta method, which were used for the power analysis.

The power function produced three correlated groups, represented by each of the observed known metabolites (‘SM(36:2)’ and ‘SM(39:1)’), and the novel potential biomarker, namely ‘PC(34:2)’. The three features achieved effect sizes of 0.74, 0.66 and 0.68, respectively. The power calculations across all three groups had a similar performance, achieving a maximal power between 35 and 45 samples (Additional file 1, SF4B). The relationship between the three subset features and the outcome variable is presented in Fig. 4b.

### Classification mode

#### Simulation data

The simulated dataset was modified to produce a binary classification outcome variable (y) and were then utilised for the feature selection (Table 3 and Additional file 1, SF5A). Within the HS environment, while all methods successfully identified all of the variables from the first group that were used to generate y (V1–V10), only a single variable from the second group (V11–V20) was identified by the Boruta and the Permutation (Corrected) methods and none of the variables were identified by RFE. Permutation (Raw) was the most successful method within the HS environment, identifying V11–V19 from the second group. No method identified any variables from the third group (V21–V30). Within the LS environment, the Boruta method exhibited the greatest stability, increasing its tally of true positive features to 29, while identifying only 9 false positive variables. The RFE, Permutation (Raw) and Permutation (Corrected) methods also increased their true positive tally to 29, and identified 201, 465 and 110 false positive variables, respectively (Table 3).

We conducted a power analysis (Additional file 1, SF5(B)) using the eleven true positive features that were stably selected by the Boruta method within the HS environment. The power function correctly identified two groups of features (V1-V10 and V11) and subsequently the Cohen’s d effect sizes for each variable was calculated. Several features were reported with equally large effect sizes and V2 was chosen at random to represent the first group. Both the V2 and V11 features exhibited effect sizes greater than the 0.8 threshold for a large effect defined by Cohen (1998)[33], with the value of 1.82 observed for V2 and 0.88 for V13. Consequently, maximum power values were achieved for both features when employing a sample size < 20.

The normal distribution simulation results are reported in the Additional file 1, SF13. In summary, we found that V1–V10 andV21–V30 were selected by all four methods with 100% frequency. Variables selected by the RFE method were also selected by the Boruta method, but not vice versa. The overall class error rate using the stable features were estimated to be 3%. The list of the classification error across other methods and data sets are listed in the Additional file 1, Table 2).

#### Dataset 3: metabolomics

To further assess the classification approach’s performance, we utilised the dataset resulting from Acharjee et al. (2016a, b [21], with a modified binary classification outcome variable, namely a below or above the mean relative liver weight value (Table 3 and Additional file 1, SF6A and SF6B). The application of the Boruta and Permutation (Raw) methods resulted in the selection of a similar number of features in both the HS and LS environments. Within the HS environment, both methods selected two, known, lipids and ~ 20 novel features of interest. Within the LS environment, the methods identified an additional known lipid alongside ~ 70 potential novel features.The RFE method identified comparatively few features within the LS environment and none within the HS one. Conversely, the application of the Permutation (Corrected) method resulted in the selection of eight HS features, more than double that the ones identified during the regression analysis. The 26 HS stable features, selected by the Boruta method, were used to conduct the power analysis. Two groups of correlated features were identified, namely ‘PC(32:0) or PE(35:0)’, with a Cohen’s d effect size of 1.92 and ‘PC-O(35:5) or PC-P(35:4)’, with an effect size of 2.24. These values exceed the Sawilowski’s descriptors of ‘very large’ and ‘huge’, respectively [41]. Maximum power values were achieved for each group at a sample size of ~ 10–20 (Additional file 1 SF6B).

#### Dataset 4: transcriptomics

Finally, we applied the binary classification approach to three different genomics cohorts. The first dataset features four healthy control samples as well as samples from stages 1–4 colorectal cancer (CRC). The analysis was conducted as a series of pairwise classifications aiming to identify the biomarkers that distinguished, most effectively, the healthy controls from samples at each CRC stage (Additional file 1, control vs stage 1 in Table 3 and other stages in SF7). Across all four pairwise comparisons, the application of the Boruta method allowed us to select a similar number of features within both the HS and LS environments. The application of the Permutation (Raw) method resulted in the selection of fewer features under HS compared to LS but remained reasonably sensitive, successfully selecting a number of HS stable features across all but the control vs stage 1 analyses. The RFE methods exhibited a low sensitivity across all comparisons, identifying a maximum of 14 LS feature but no HS features. Similarly, the Permutation (Raw) method identified a limited number of HS features and the second largest number of LS features, indicating a poor selection stability between iterations.

The second genomics data was reported by Quraishi et al. [37] with the aim to identify differential genes between patients with PSC have colitis (PSC-IBD) vs. ulcerative colitis (UC). The analysis was conducted using the same framework and a total of 59 stable features (Additional file 1, SF10) were identified. Out of 59 genes that were identified, 15 genes were also reported by Quraishi et al. [37].

The third dataset resulted from a comprehensive lipidomic and transcriptomics study of white adipose tissue in mice that become obese either via a genetic modification (ob/ob) or diet (high fat diet) [38]. The genotype class:OB/OB type vs. wild type was considered as the outcome variable (Additional file 1, SF11). The application of the Boruta method allowed us to identify 101 stable features from the gene expression datasets.

The HS stable features, selected by the Boruta method, for each of the four analyses, formed the binary classification power functions (Module 2). For each case, these features were converted into correlated groups and the feature, from each group, with the largest Cohen’s d effect size, was determined. In addition, for all cases, statistically significant differences, based on t-tests conducted for each feature, were observed between binary classes (Additional file 1, SF8).

The conducted power calculations determined the three features identified in the control vs stage one. Using these three features, a maximum power can be achieved using < 10 case samples. In other cases, the required number of samples vary from 5 to 1280 (Additional file 1, SF9). In each comparison scenario, the effect size of all features was greater than the 1.0 threshold [39].

#### Web tool

**Fig. 5:**
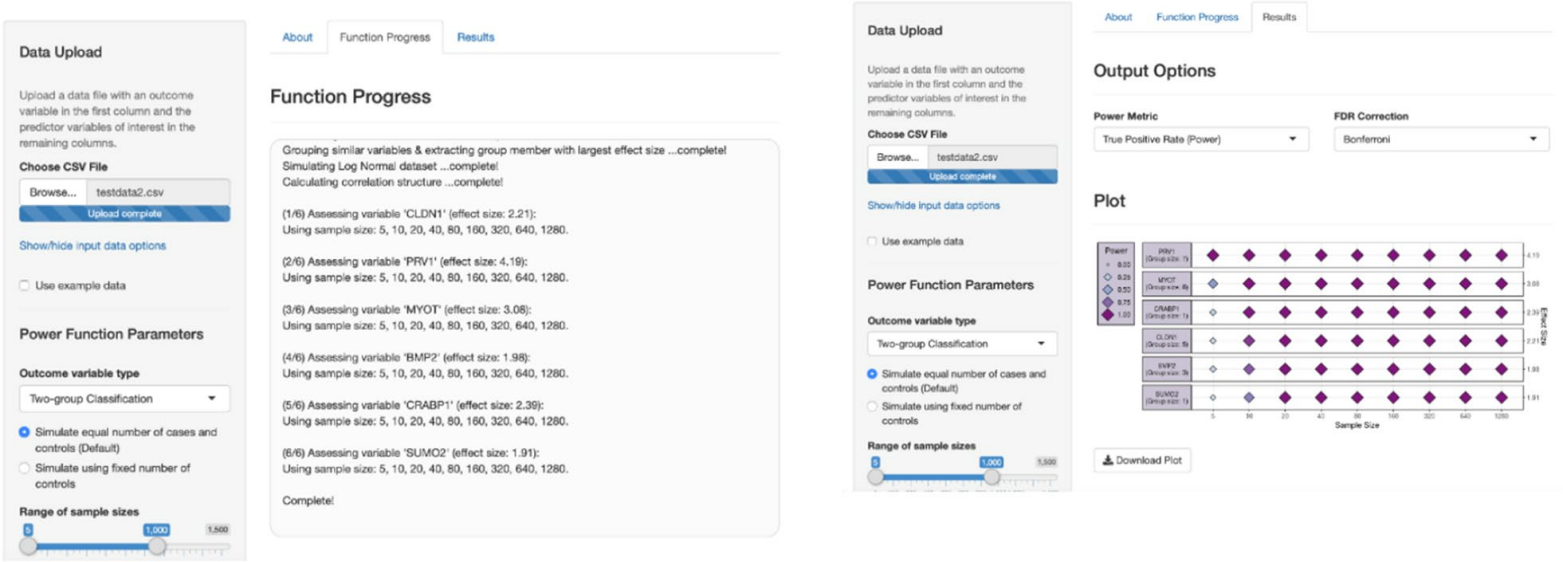
Screenshots of the open- source web application ‘PowerTools’, for efficient and accessible simulation based power calculations

To streamline the power calculation estimation, we produced PowerTools, an interactive open-source web application, written in R code, using the Shiny framework (http://shiny.rstudio.com/) (Fig. 5). The tool uses different -omics types as input and performs efficient simulation-based power calculations for regression and classification analysis as well as for two-group classification. The web interface allows for a simple sample size specification as well as the visualization of the graphical output. The confusion matrix result values are presented as downloadable customisable plots as well as raw data tables. A user guide is provided in the Additional file 2.

## Discussion

We have assessed the performance of four RF feature selection methods in the context of regression and classification, using both simulated and published experimental datasets. The known underlying correlation structure and relationship with the simulated data outcome variable (y) was used for the method assessment in terms of the number of true and false positive features each method identified.

In the regression context, all methods were stable in their selection of true positive features within the HS environment, whereas the Boruta method alone maintained near perfect specificity within the LS environment. In contrast, the application of the Permutation (Raw) method resulted in the identification of an additional 98 false positive features with the LS environment in comparison to the ones identified within the HS one. These findings suggest that multiple iterations of feature selection, combined with a high stringency threshold for stability, effectively eliminate any false positive features selected by these methods by chance. The high feature stability obtained by the Boruta method was characterised by the smallest difference in performance between stringency contexts, suggesting that its application might be favoured in scenarios where the large computational runtime multiple iterations necessitate cannot be guaranteed.

When we applied RF based feature selection methods to publicly available data, it was impossible to distinguish variables truly associated with the outcome from false positives. Consequently, the subset of features, selected by each method, were assessed only in terms of their stability, numerosity and the predictive performance of their resultant validation models. Across the two published experimental datasets considered for regression, the Boruta method exhibited the best stability levels, producing the smallest difference in the number of the selected features between the different stringency states. In addition, the Boruta method identified the largest number of stable HS features across multiple datasets producing higher numbers of biomarker candidates [42].

The RFE method identified the smallest subset of HS stable features for every dataset that it was assessed against, a finding that was expected since the method is designed to identify the smallest subset of predictive features [27]. Consequently, the RFE method exhibited the worst stability, producing a 15-fold difference in the number HS/LS features identified for the metabolomics data set and an 11-fold difference for the lipidomics one. Recently, [43] discussed a heuristic method for the identification of biomarkers, termed RGIFE (Rank Guided Iterative Feature Elimination), which is a variant of the RFE method. RGIFE employs information, extracted from machine learning models, to ensure that the minimal and highly predictive marker sets can be established.

In terms of classification, each method resulted in fewer stable feature selection for both the simulated and the experimental datasets. However, each of the method that was applied had a similar performance and the Boruta method exhibited the highest stability, across all cases. The assessment of the methods’ performance over simulated data revealed that all methods were able to identify only true positive variables within the HS environment, and the Permutation (Raw) method identified the highest number of true positive variables.

One of the limitation of the Boruta method lies with the time complexity associated with its performance when dealing with large high dimensional datasets. [26] For the case of the metabolomics dataset, the Boruta and Permutation (Raw) methods exhibited a similar stability, while the Boruta method identified the largest number of biomarker candidates. For the case of the cancer transcriptomics dataset, Boruta exhibited a similar performance between each pairwise comparison of controls and CRC stage. The Boruta method selected HS features across all four comparisons and exhibited a consistent selection behavior within the LS environment. RFE, on the other hand, exhibited the poorest stability in the case of the IBD and mice gene expression datasets, failing to select a single HS feature across them. Overall, however, across all experimental datasets that were assessed, our approach was successful in retrieving the majority of the known metabolites or genes indicating its potential for identifying important features that can facilitate potential novel biomarker discovery.

The assessment of the RF model’s performance across the different datasets revealed limited variation, a finding which is supported by various previous studies [42, 44]. The RF algorithm is capable of compensating for noisy features with limited model performance loss [44, 45]. Furthermore, the variance in predictive performance values between CV repeats was greater when using variables selected within the HS environment (e.g. Fig. 2c). This effect can be understood with respect to the greater differences, between outer loop repeats, in the variables meeting the HS selection criteria, compared to the equivalent differences in those which surpass the LS selection criteria.

One of the limitations of our approach lies with our focus on the use of feature solemnly selected by RF. Any classifier that can provide a ranking of feature’s importance would be suitable for our framework. RF, however, has been reported as being potential advantageous over other classification approaches. For example, Couronné et al., [46] used a large-scale benchmarking experiment, based on 243 experimental datasets, comparing the prediction performance of the RF and logistic regression. They reported RF to perform better than logistic regression in terms of the accuracy measured (in approximately 69% of the datasets). The use of RF over support vector machine (SVM) is also supported by several studies. For example, in a cancer related study [47], electronic tongue data classification RF [48]) was reported to have a consistently good performance.

We sought to combine our stable feature selection protocol with a novel simulation-based approach to facilitate power calculations for the design of potential future studies. Further to previous efforts by [24], who compared the power achieved by a multitude of classifiers when presented with diverse sets of data, we focused on RF but expanded into both the classification and regression domains. Furthermore, we ensured that the true correlation structure of the input data was captured by applying a methodology originally used in the context of metabolic phenotyping [23]. We incorporated automated effect size calculations, and grouped similar variables before filtering them by effect size, to identify a small subgroup of high effect putative biomarkers for which to quantify power.

We validated the performance of our power calculations with respect to the values achieved using the simulated regression data. The power values predicted for each feature matched the observed empirical power achieved by most stable feature selection methods (Additional file 1, SF2B). Furthermore, as expected, we consistently observed that smaller necessary sample sizes are required for features with larger effect values, An example of this effect is illustrated for the case of the CRC stage 3 data [36] (Additional file 1, SF9C), where the effect sizes ranged between 4.78 and 1.65, and the sample size necessary to obtain maximal power ranged from < 5 to > 1280. We also observed that the Boruta method, in terms of its prediction ability, exhibited the best performance in contrast to the RFE which had the worst performance primarily due it selection of few features (Additional file 1, Table 1 and 2).

We explored a variety of sample sizes so as to identify the necessary ones to achieve good power, concluding that less than 40 samples is necessary to achieve maximum power with an effect size of 0.8 in a regression mode and no more than 10 samples is necessary, with a Cohen’s d effect of 3.0, in a classification mode. The effect sizes reported are consistent amongst the stable features selected by Boruta. For almost all the cases assessed, fewer samples were necessary to achieve maximum power for feature selection during classification than regression. This observation corroborates the high stringency observed for the classification mode feature selection in module one.

Lastly, we developed PowerTools, an interactive open-source web application, to facilitate the estimation of the number of samples required for potential future studies and to cater the clustering of similar features determining the effect size associated with potential biomarkers. While other power analysis tools exist, most have different functionality limitations, for example providing only raw functions [23, 49], relating only to specific study designs, such as case–control microbiome studies [50], or lacking support and further development [24]. We believe that our workflow overcomes such limitations and is generalised across multiple different –omics datasets aiding the translational community’s efforts to interpret the stability of novel biomarkers and design potential future validations studies.

## Conclusion

In this paper, we presented a number of different RF based stable feature selection methods and compared their performances using simulated as well as publicly available experimentally derived datasets. Across all of the scenarios considered, the application of the Boruta method formed the most stable approach, while the application of the Permutation (Raw) method, when allowed to be stabilised over a number of iterations, resulted in the identification of the largest number of relevant features. We determined that the decision over the approach selection should be weighed against the computational requirements and the runtime requirements. Finally, we have developed a web-based interface that caters the calculation of the effect size of the stable biomarkers for potential future studies.

## Supplementary information

**Additional file 1**. Random Forest methods, figures (SF1-SF13) and tables (1 and 2) on selection, ranking, hyperparameter optimization are provided.

**Additional file 2**. A user guide on PowerTools.

## Data Availability

The datasets analysed during the current study are available in the https://github.com/joelarkman/RF-FeatureSelection-PowerAnalysis repository.

## Abbreviations

ANOVA: Analysis of variance
CBGS: Cambridge baby growth study
CV: Cross validation
HS: High stringency
MSE: Mean squared error
Ob/Ob: Obese mice mutation
OOB: Out-of-bag
LS: Low stringency
PC: Phosphatidylcholine
PSC: Primary sclerosing cholangitis
RF: Random forest
RFE: Recursive feature elimination
RGIFE: Rank guided iterative feature elimination
R^2^: Predictive power
SM: Sphingomyelin
UC: Ulcerative colitis
