# Supplementary material for: A random forest based biomarker discovery and power analysis framework for diagnostics research
Source: BMC Med Genomics. 2020 Nov 23;13:178. doi: 10.1186/s12920-020-00826-6 (PMC7685541; doi:10.1186/s12920-020-00826-6)
Supplement: Supplementary file 1 — Additional file 1. Random Forest methods, figures (SF1-SF13) and tables (1 and 2) on selection, ranking, hyperparameter optimization are provided. [file 12920_2020_826_MOESM1_ESM.docx]

**Title: A Random Forest based biomarker discovery and power analysis framework for diagnostics research**

**Animesh Acharjee^1,2,3,*,#^, Joseph Larkman^1,2,*^, Yuanwei Xu^1,2^, Victor Roth Cardoso^1,2,4^, Georgios V. Gkoutos^1-6^**

^1^College of Medical and Dental Sciences, Institute of Cancer and Genomic

Sciences, Centre for Computational Biology, University of Birmingham, B15 2TT, UK

^2^Institute of Translational Medicine, University Hospitals Birmingham NHS, Foundation Trust, B15 2TT, UK

^3^NIHR Surgical Reconstruction and Microbiology Research Centre, University Hospital Birmingham, Birmingham B15 2WB, UK.

^4^MRC Health Data Research UK (HDR UK)

^5^NIHR Experimental Cancer Medicine Centre, B15 2TT, Birmingham, UK

^6^NIHR Biomedical Research Centre, University Hospital Birmingham, Birmingham, B15 2TT, UK.

*** Sharing First authorship**

**# Corresponding Author.**

**Animesh Acharjee**

**Tel.: +44 (0)1213718135**

**E-mail: a.acharjee@bham.ac.uk**

**SUPPLEMENTARY METHODS**

***RF Variable Importance***

The permutation importance of a particular variable is an importance metric calculated as the difference in RF prediction performance before and after permutation of the variable’s values, averaged across all constituent trees. Variables that largely impact upon model performance will have large permutation importance values, whilst variables with no relevance will produce a value close to zero. In this way, RFs can be used to establish a ranked list of variables according to their observed importance. Both the standard RF implementation from the R package randomForest (1) and an optimised, more memory efficient implementation from the R package ranger (2) were utilised here. The implementation used for each feature selection method was dependent on their default methodology.

***Hyperparameter Selection***

Three hyperparameter values must be specified prior to execution of the random forest algorithm: (a) the number of variables to compare at each decision tree split (*mtry*), (b) the number of constituent trees to generate for the forest (*ntree*), and (c) the minimum size of the terminal nodes of each tree (*nodesize*). In order to identify an optimised suite of values for each of the data sets used in the present study, a portion of cross-validation data was subset for hyper parameter optimisation. The data was then subject to the iterative training of RFs using a broad range of parameter values designed to scope either side of their respective default values. The performance at each combination of parameter values were then compared and visualised in terms of the estimated accuracy (R squared) of the resultant model. This process was facilitated using the train function from the R library ‘Caret’ (3). We produced a modified training method that accepted a range of values for all three parameters with which to assess model performance. Consequently, *ntree* was increased from its default of 500 and fixed at 2000; *nodesizes* were held at their default of one for the classification case and five for regression forests; and *mtry* values were set to the square root of the number of variables provided. This final suite of parameters achieved both strong modelling performance and a reasonable computational runtime.

**Power analysis**

The functions described by Blaise et al., (2016) (4) were written in MATLAB^TM^ and had limited accompanying documentation. Here, we reimplemented their base functionality in an R environment, increasing their accessibility. Data simulation from the multivariate log-normal distribution was facilitated via the ‘mvrnorm’ function from the R library MASS, whilst correlation, one-way ANOVA, and regression calculations, were facilitated by functions from the base R Stats library. Additionally, the redesigned functions incorporated comprehensive progress messages and error notation, to improve their ease of use. Furthermore, the R implementation presented here builds upon the functionality of the original functions in two key respects: (a) Each variable is automatically assessed using its true effect size; in the case of regression, the true effect size of a variable is estimated as its correlation with the true outcome variable, whilst for two-group classification, the observed Cohen’s d effect size (5) is computed; (b) Highly correlated variables can optionally be grouped together and only the member of each group with the largest effect size used for assessment, therefore, facilitating the identification of a smaller subset of potential biomarkers.

***SUPPLEMENTARY FIGURES***

***
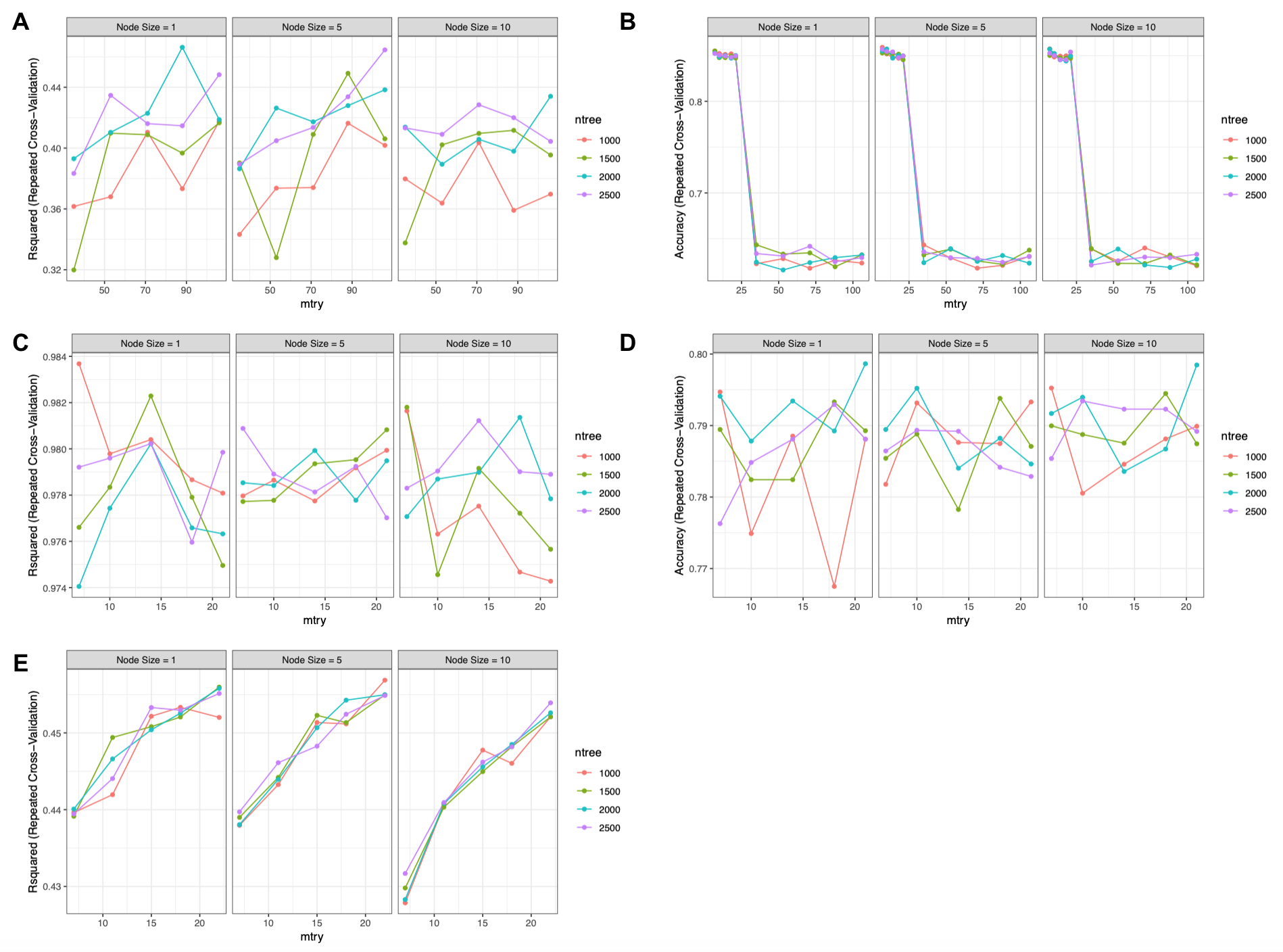
***

***Supplementary Figure 1 (SF1): Results from hyperparameter optimisation. (A)*** *Simulation data, regression mode.* ***(B)*** *Simulation data, classification mode.* ***(C)*** *Real data 1* (6)*, regression mode.* ***(D)*** *Real data 1* (6)*, classification mode.* ***(E)*** *Real data 2* (7)*. In each subfigure R squared performance metrics are displayed along the y axis for RF models trained using the inner test data partition of the relevant data set. Facets within each plot denote the values achieved using three different node sizes; line/point colours specify the performance achieved across four ntree values; while along the x axis performance values are compared using a value 0.5, 0.75, 1.0, 1.25 and 1.5 times the default value for mtry (square root of the number of variables).*


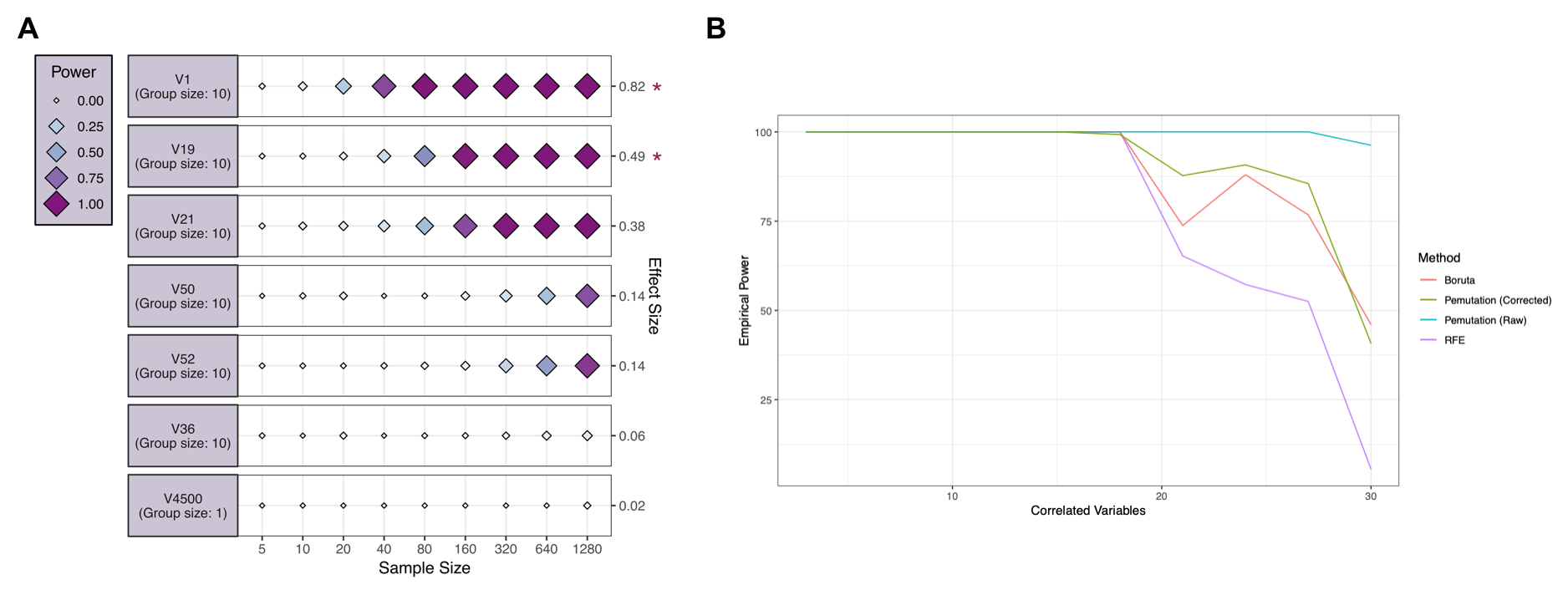


***Supplementary figure 2 (SF2): Power analysis of simulation data (regression mode). (A)*** *The eight groups of correlated features are represented by the group member with the largest observed effect size. The effect size of each assessed variable is shown along the y axis and a series of sample sizes along the x axis. The name of each assessed variable is shown alongside the number of similar (grouped) features. Power values determined for each effect/sample size combination using a simulated dataset with the same correlation structure as input data and displayed using variably sized/coloured rhombi. The HS stable features selected by Boruta are indicated with an asterisk.* ***(B)*** *The frequency with which each of the thirty true causal variables were selected by each feature selection method after 100 iterations. Values are shown as the average frequencies across four outer-loop cross-validation repeats.*


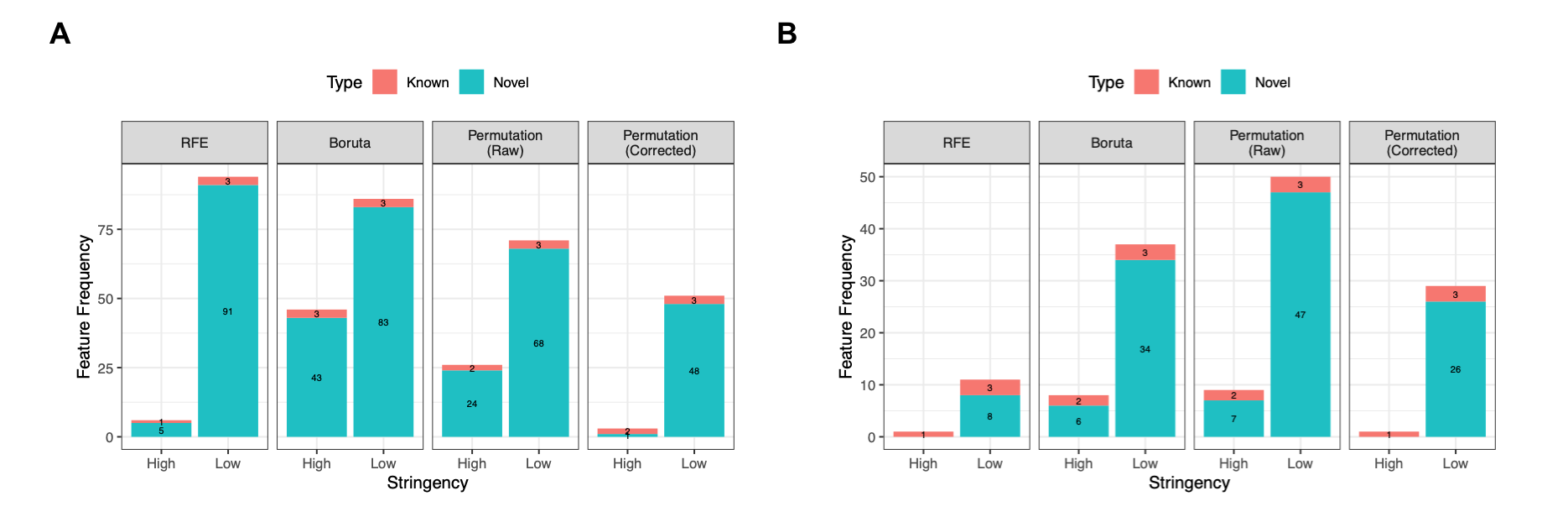


***Supplementary figure 3 (SF3): Feature selection results for (A) real data 1, regression mode*** (6) ***and (B) real data 2, regression mode*** (7)***.*** *Stable features are defined in terms of those selected by each approach in at least 5/100 iterations (Low Stringency) or a minimum of 90/100 iterations (High Stringency). Features previously identified in the literature are denoted in red as ‘known’, whilst the number of novel features are shown in blue. Values describing the number of times each feature is chosen by a particular approach are averaged across those achieved after 100 iterations for each of the four inner loop test datasets.*


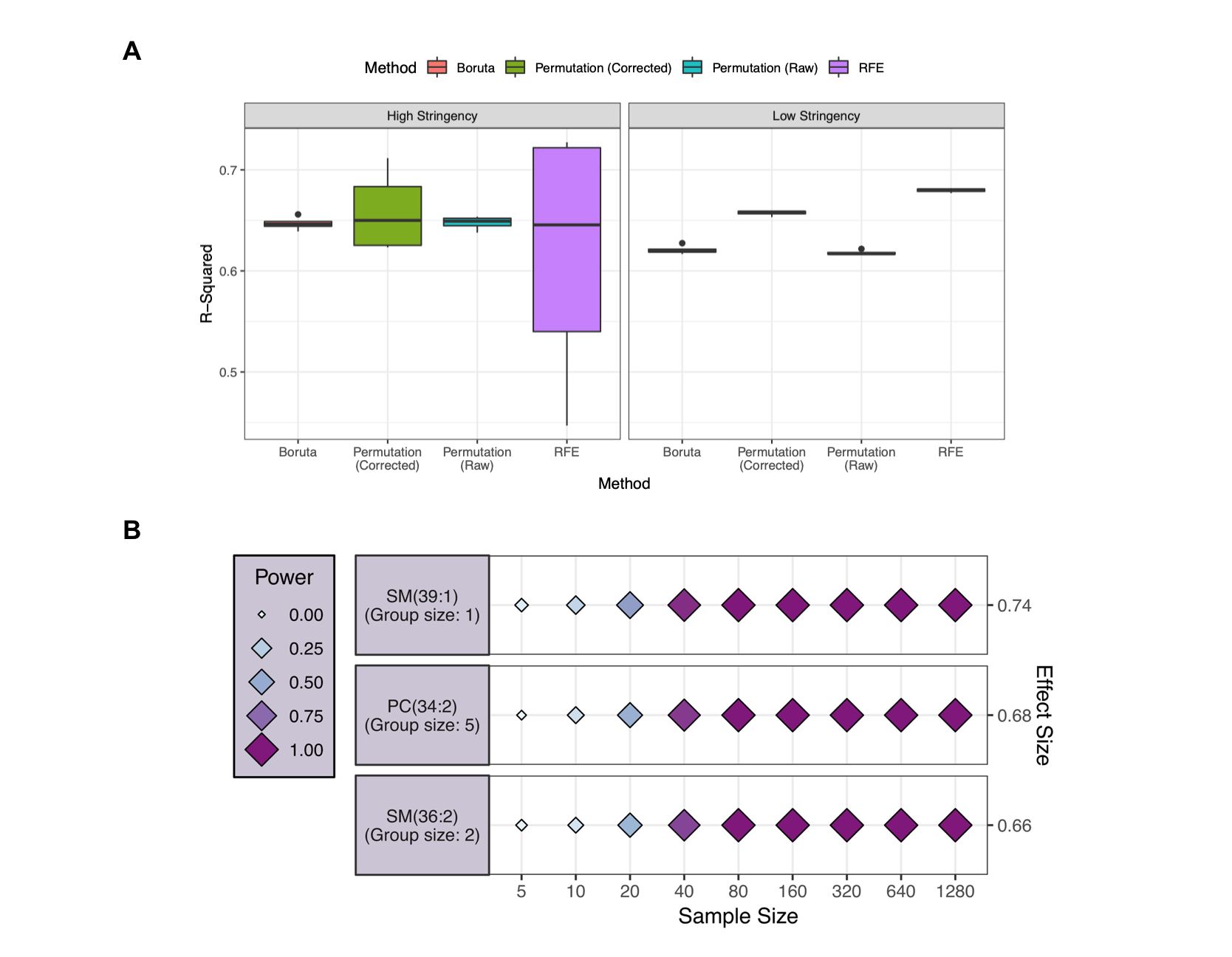


***Supplementary figure 4 (SF4): Validation model performance and power analysis of real data 2, regression mode*** (7)***. (A)*** *Boxplots displaying the variance in the observed R-squared value of validation models trained using the stable features selected by each feature selection approach, across four outer-loop CV repeats. True positive: V1-V30, False positive: V3-V5000. Values are shown for models trained using either the features selected by each approach in at least 5/100 iterations (Low Stringency) or a minimum of 90/100 iterations (High Stringency).* ***(B)*** *The three groups of correlated features identified by the power function are represented by the group member with the largest observed effect size. The effect size of each assessed variable is shown along the y axis and a series of sample sizes along the x axis. The name of each assessed variable is shown alongside the number of similar (grouped) features. Power values determined for each effect/sample size combination using a simulated dataset with the same correlation structure as input data and displayed using variably sized/coloured rhombi.*

*
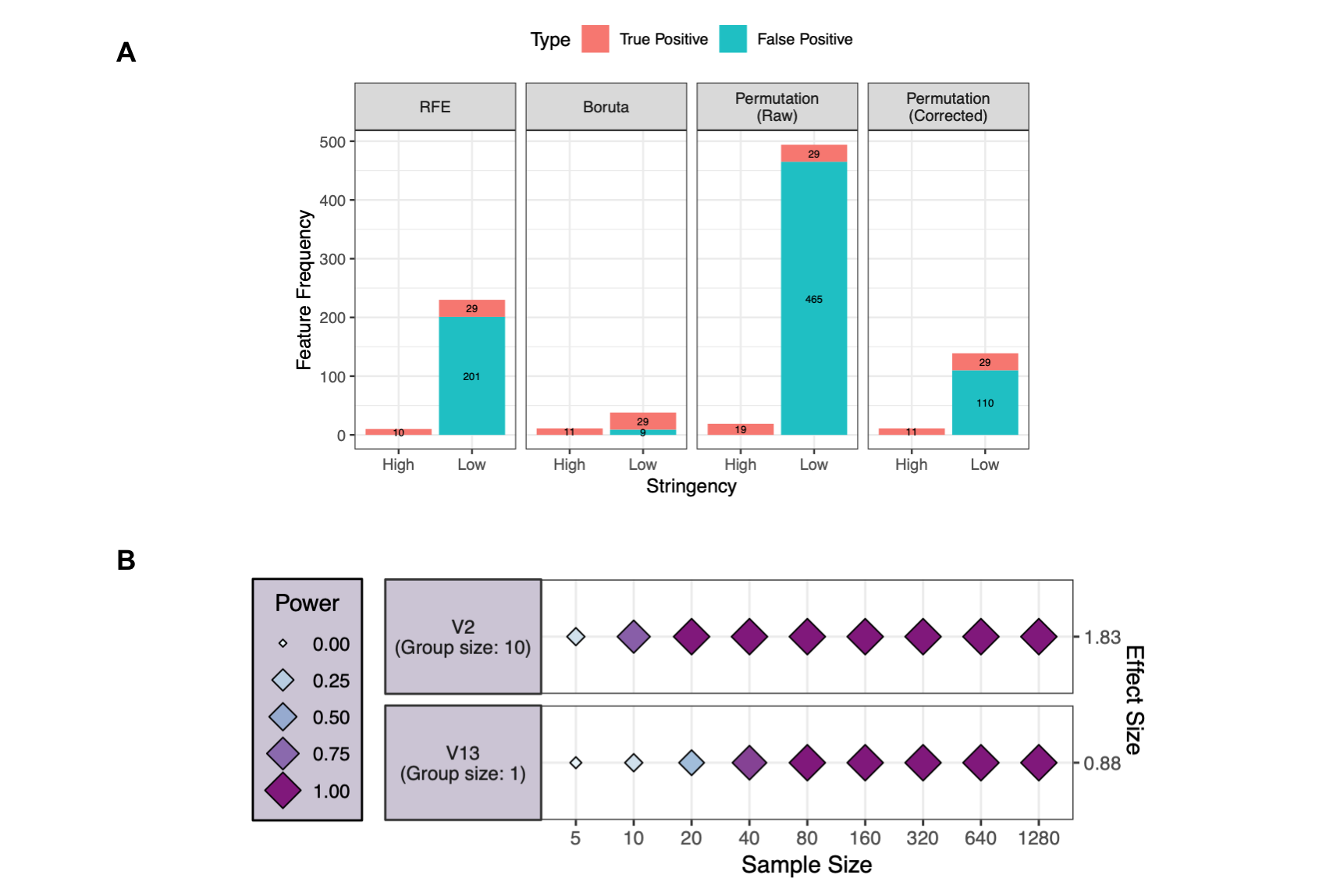
***Supplementary figure 5 (SF5): Feature selection and power analysis of the simulated data, classification mode. *(A)*** *Stable features are defined in terms of those selected by each approach in at least 5/100 iterations (Low Stringency) or a minimum of 90/100 iterations (High Stringency). Values describing the number of times each feature is chosen by a particular approach are averaged across those achieved after 100 iterations for each of the four inner loop test datasets.* ***(B)*** *Two groups of correlated features are represented by the group member with the largest observed effect size. The effect size of each assessed variable is shown along the y axis and a series of sample sizes along the x axis. The variable number of each of those assessed is shown alongside the number of similar (grouped) features. Power values determined for each effect/sample size combination using a simulated dataset with the same correlation structure as input data and are displayed using variably sized/coloured rhombi.*


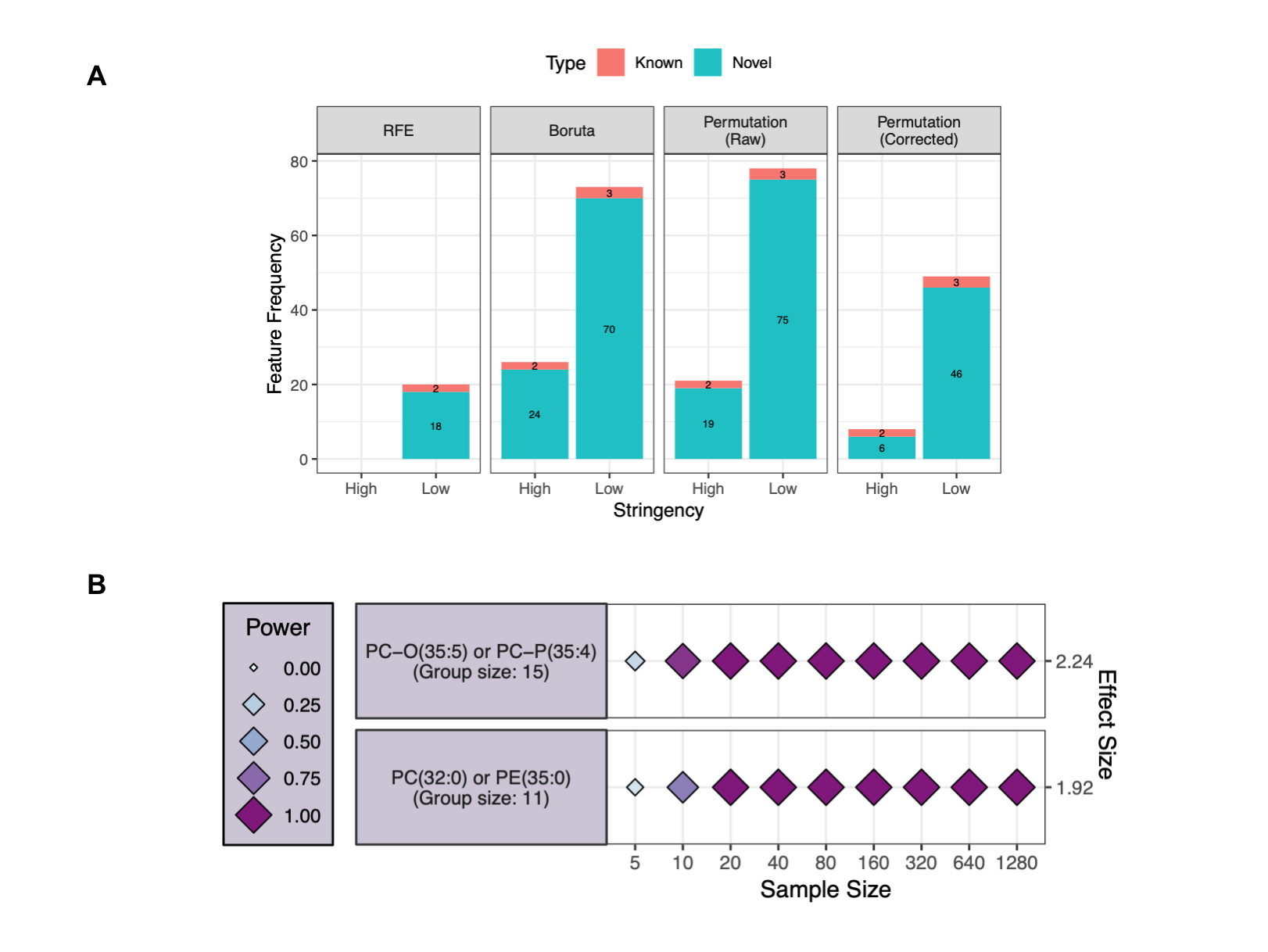


**Supplementary figure 6 (SF6): Feature selection and power analysis of real data 1, classification mode** (6)***. (A)*** *Stable features are defined in terms of those selected by each approach in at least 5/100 iterations (Low Stringency) or a minimum of 90/100 iterations (High Stringency). Values describing the number of times each feature is chosen by a particular approach are averaged across those achieved after 100 iterations for each of the four inner loop test datasets.* ***(B)*** *Two groups of correlated features are represented by the group member with the largest observed effect size. The effect size of each assessed variable is shown along the y axis and a series of sample sizes along the x axis. The name of each assessed variable is shown alongside the number of similar (grouped) features. Power values determined for each effect/sample size combination using a simulated dataset with the same correlation structure as input data and are displayed using variably sized/coloured rhombi.*


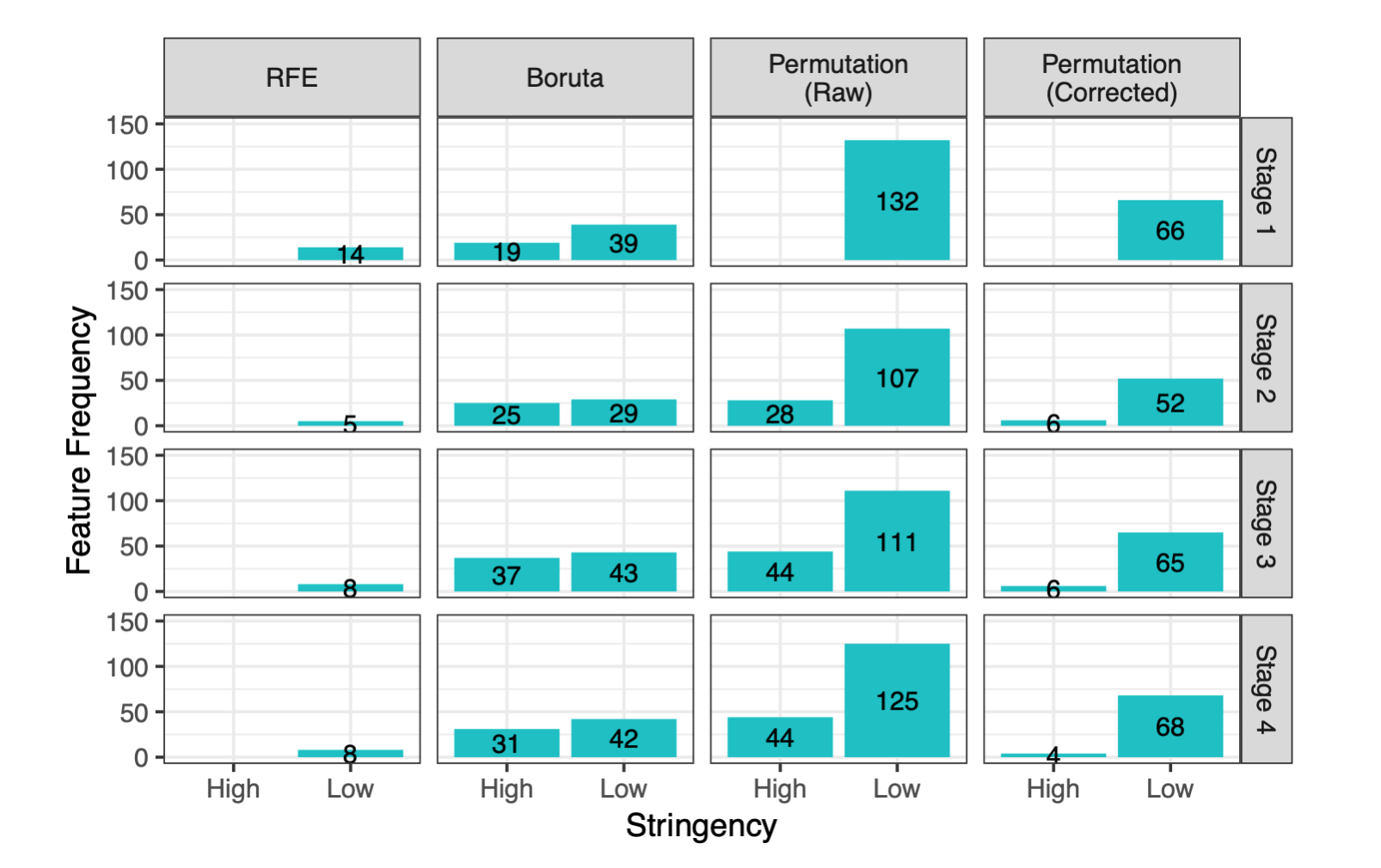


***Supplementary figure 7 (SF7): Feature selection results for the second real classification dataset*** (8)***.*** *Results are shown from each two-group classification procedure, where features distinguishing each CRC stage from four healthy controls were sought. Stable features are defined in terms of those selected by each approach in at least 5/100 iterations (Low Stringency) or a minimum of 90/100 iterations (High Stringency). Values describing the number of times each feature is chosen by a particular approach are averaged across those achieved after 100 iterations for each of the four inner loop test datasets.*

**
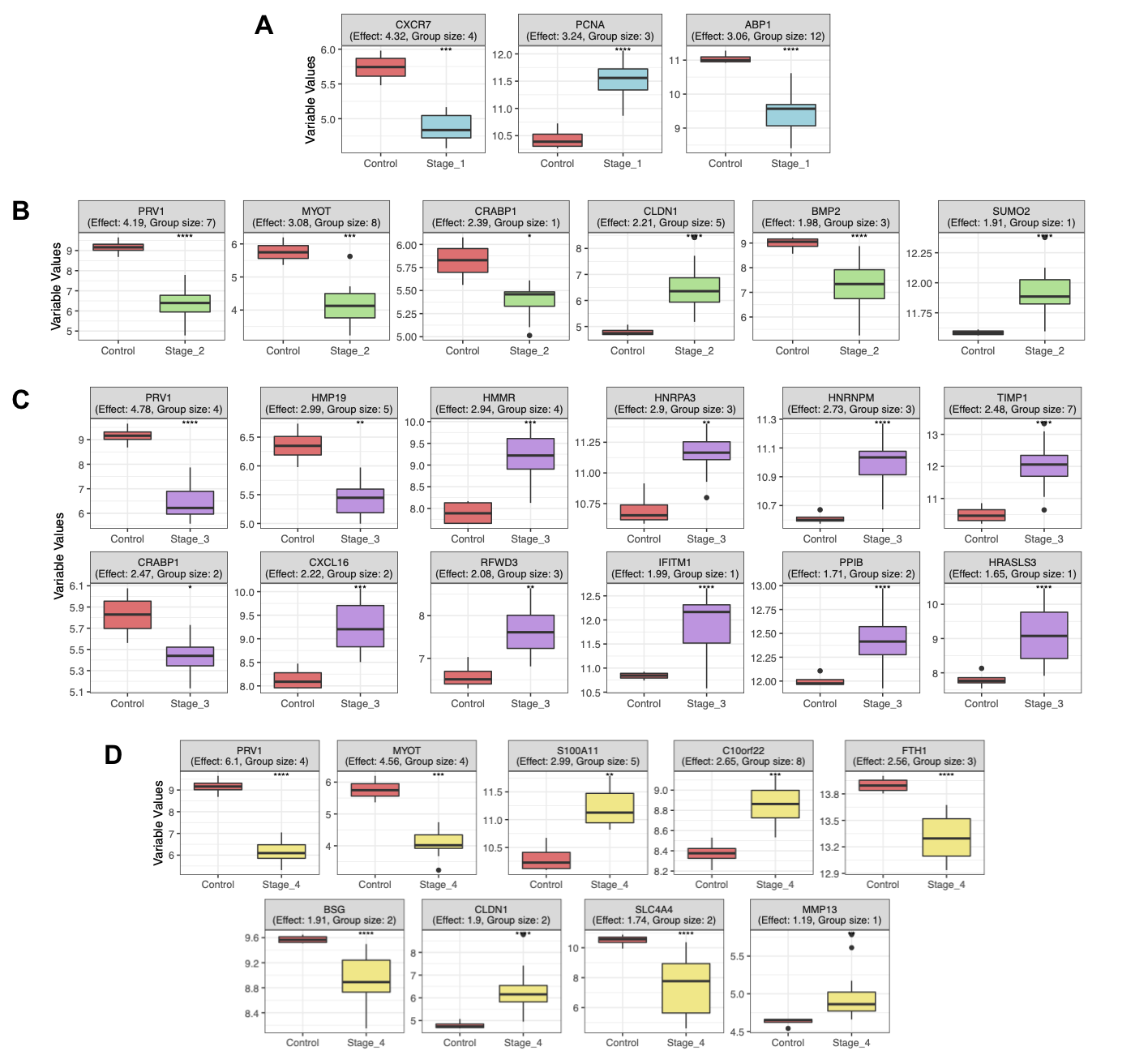
**

***Supplementary figure 8 (SF8): Groups of Up and down regulated genes from each cancer stage selected by module 1 of the workflow and grouped by module 2 power functions.*** *Each group is represented by the gene with the largest observed Cohen’s d effect size. Gene name, effect size and group size values are displayed above each subplot. Expression values statistically evaluated between each stage and controls via t-test and significance is indicated using the following convention: *:p<0.05, **:p<0.01, ***:p<0.01, and ****:p<0.0001.*


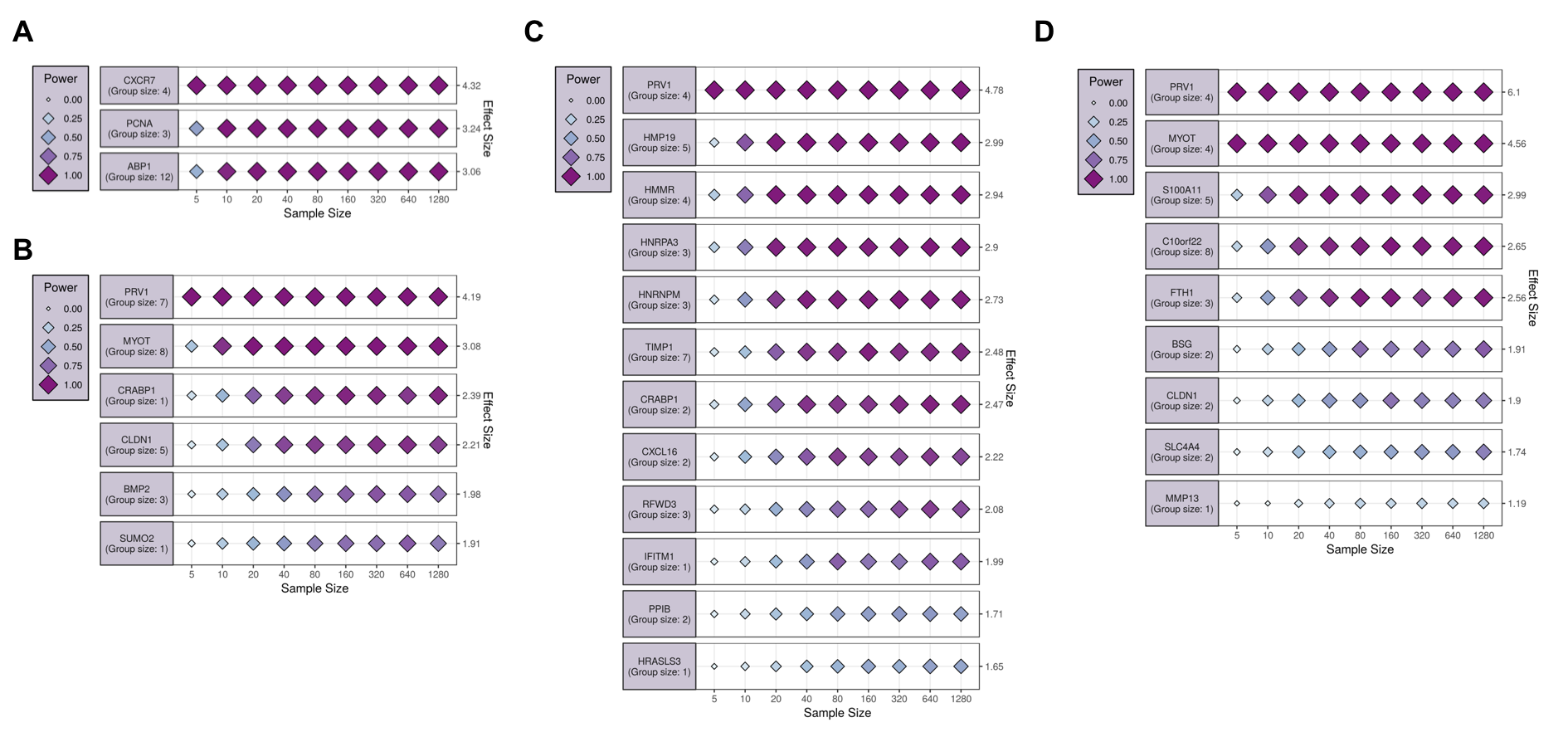


***Supplementary figure 9 (SF9): Power calculation results for the second real classification dataset*** (8)***.*** *Only the HS stable features selected for each cancer stage using Boruta, were provided to the power functions.* ***(A)*** *Control vs Stage 1,* ***(B)*** *Control vs Stage 2,* ***(C)*** *Control vs Stage 3, and (****D)*** *Control vs Stage 4. In each subfigure, groups of correlated features are represented by the group member with the largest observed effect size. The effect size of each assessed variable is shown along the y axis and a series of sample sizes along the x axis. The name of each assessed variable is shown alongside the number of similar (grouped) features. Power values are determined for each effect/sample size combination using a simulated dataset with the same correlation structure as input data and are displayed using variably sized/coloured rhombi.*


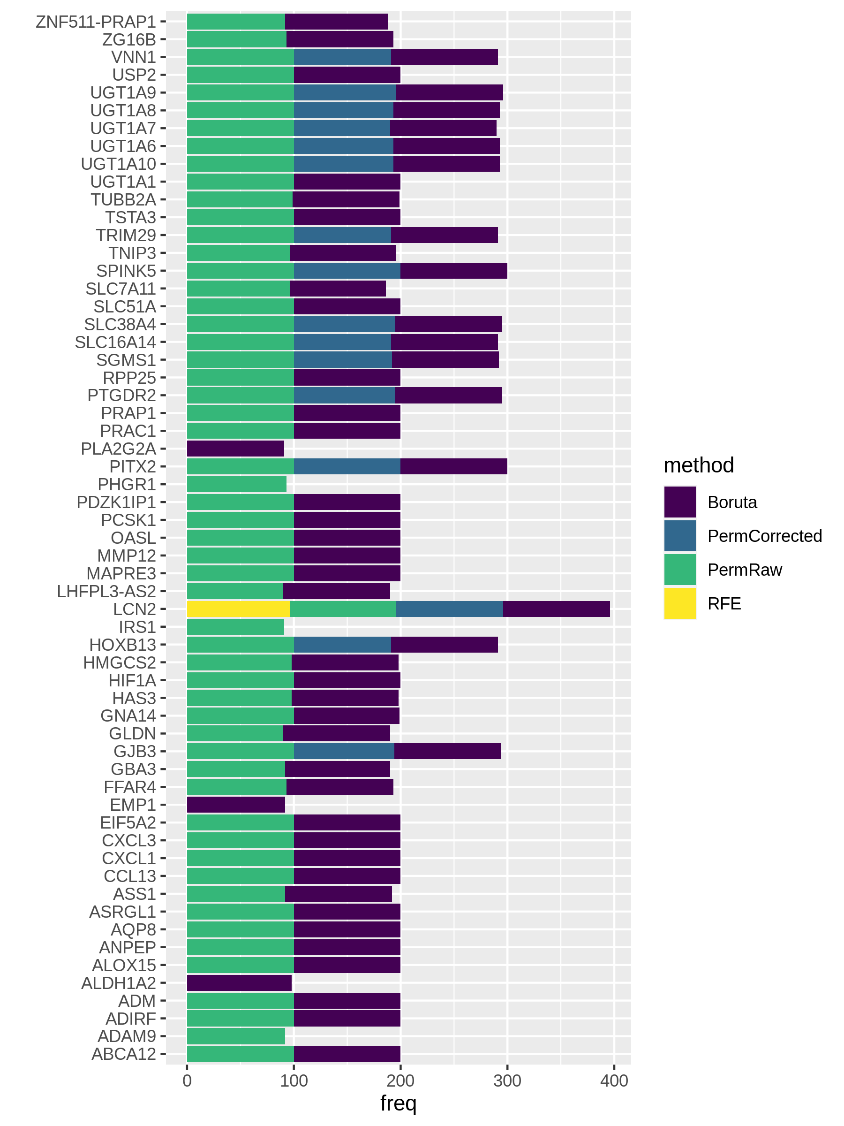


***Supplementary figure 10 (SF10):*** *Total 59 stable features are selected based on the number of frequency from the multiple RF based methods.*


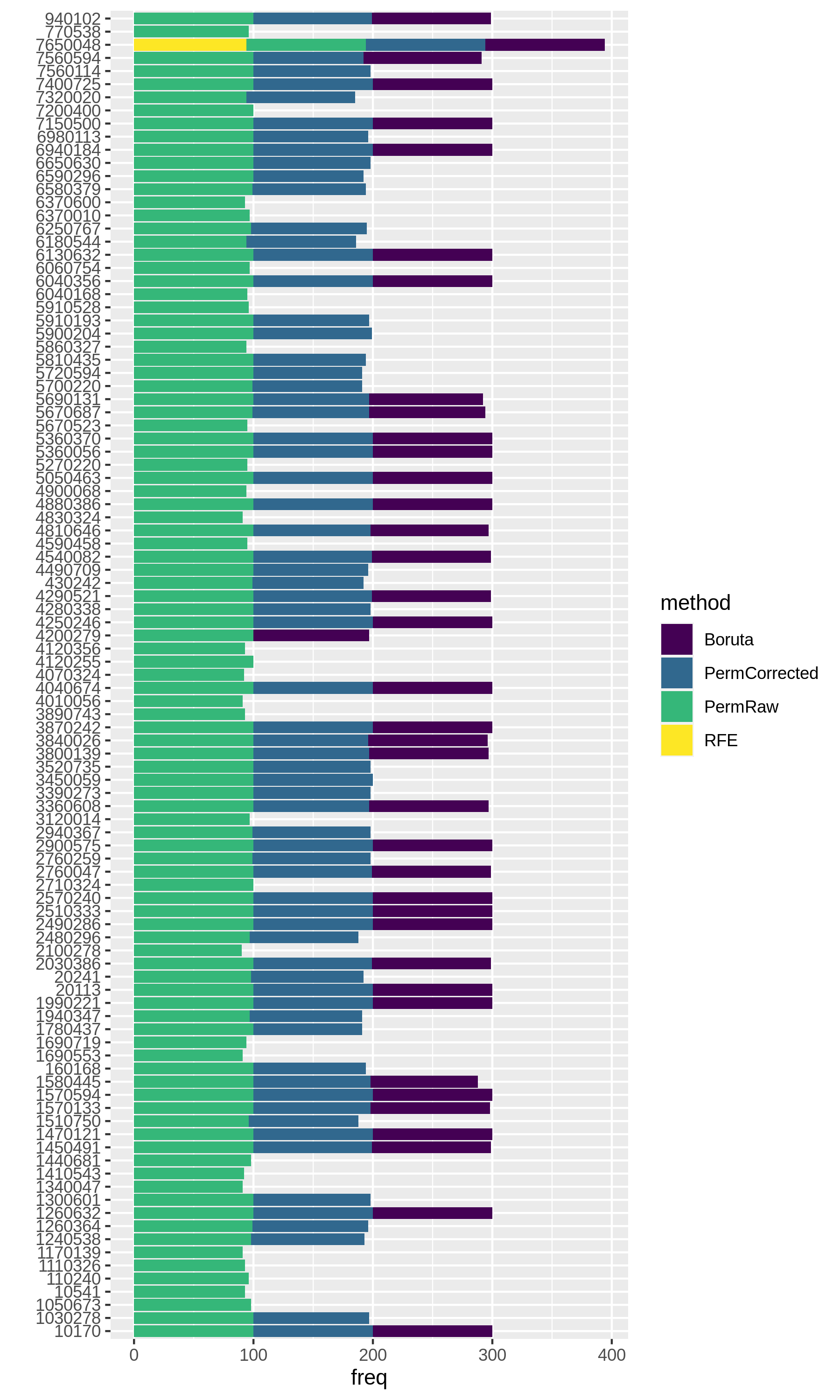


***Supplementary figure 11 (SF11):*** *Total 101 features selected for 4 RF based feature selection methods. For each feature, frequency of selection is at least 90 out of 100 iterations.*


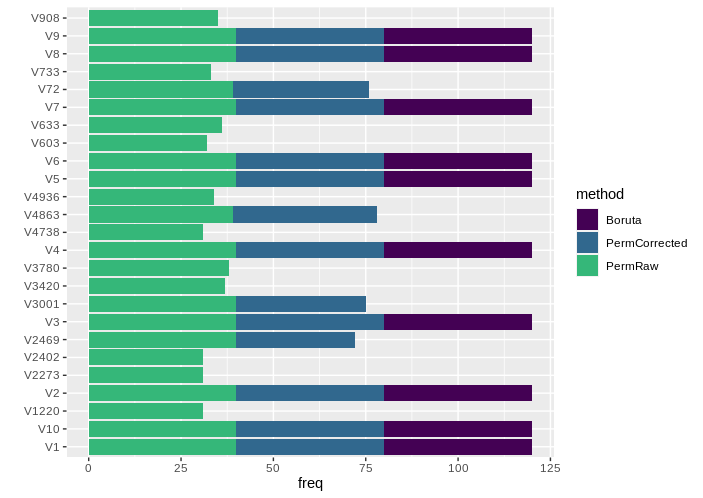


***Supplementary figure 12 (SF12):*** *Total 25 features selected for the regression task of simulated data. For each feature, frequency of selection is at least 30 out of 40 iterations. RFE did not select any of the features with a frequency of at least 30 with this method.*

***Supplementary figure 13 (SF13):*** *
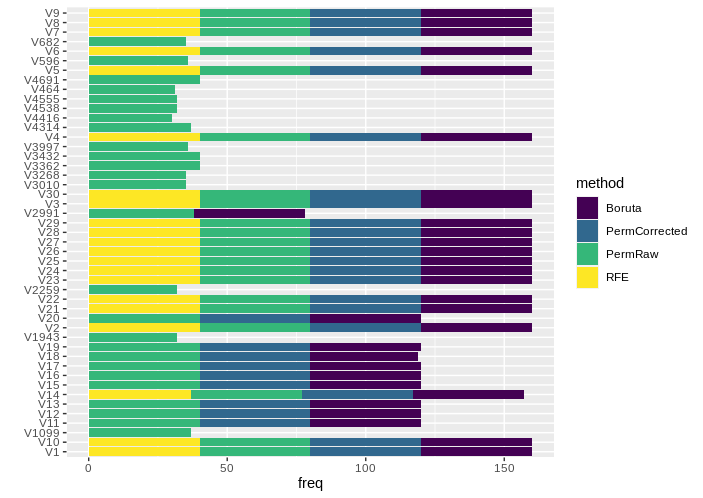
Simulated data classification 47 features selected for the classification task of simulated data. For each feature, frequency of selection is at least 30 out of 40 iterations.*

**Table 1: List of the methods and their performance on the different data sets in the regression mode and variation explained (R^2^)%.**

| **Methods** | **Synthetic data** | **Data set 1 (Metabolomics)** | **Data set 2 (Transcriptomics)** |
| --- | --- | --- | --- |
|  |  |  |  |
| Boruta | 98.22% | 95% | 93.32% |
| PermCorrected | 79% | 89% | 74% |
| PermRaw | 82.20% | 84% | 65% |
| RFE | 0% | 32% | 67% |

**Table 2: List of the methods and their performance on the different data sets as classification model and the class error for each of the outcome classes**

| **Methods** | **Synthetic data** | **Data set 1 (Metabolomics)** | **Data set 2 (Transcriptomics)** | **Data set 3 (Transcriptomics)** | **Data set 4 (Transcriptomics)** |
| --- | --- | --- | --- | --- | --- |
| Boruta | 3% | 2% | 4.23% | 5% | 10% |
| PermCorrected | 10.54% | 5.90% | 18.40% | 22% | 23% |
| PermRaw | 14.60% | 11.30% | 29.40% | 31.20% | 38.20% |
| RFE | 18% | 43.70% | 61% | 75% | 83.33% |

**REFERENCES**

1. Breiman,L. (2001) Random Forests. *Mach. Learn.*, 45, 5–32.

2. Wright,M.N. and Ziegler,A. (2017) ranger : A Fast Implementation of Random Forests for High Dimensional Data in C++ and R. *J. Stat. Softw.*, 77.

3. Kuhn,M. (2008) Building Predictive Models in R Using the caret Package. *J. Stat. Softw.*, 28.

4. Blaise,B.J., Correia,G., Tin,A., Young,J.H., Vergnaud,A.-C., Lewis,M., Pearce,J.T.M., Elliott,P., Nicholson,J.K., Holmes,E., *et al.* (2016) Power Analysis and Sample Size Determination in Metabolic Phenotyping. *Anal. Chem.*, 88, 5179–5188.

5. Cohen,J. (1988) Statistical Power Analysis for the Behavioral Sciences. Taylor and Francis, Hoboken.

6. Acharjee,A., Ament,Z., West,J.A., Stanley,E. and Griffin,J.L. (2016) Integration of metabolomics, lipidomics and clinical data using a machine learning method. *BMC Bioinformatics*, 17, 440.

7. Acharjee,A., Prentice,P., Acerini,C., Smith,J., Hughes,I.A., Ong,K., Griffin,J.L., Dunger,D. and Koulman,A. (2017) The translation of lipid profiles to nutritional biomarkers in the study of infant metabolism. *Metabolomics*, 13, 25.

8. Chen,X., Deane,N.G., Lewis,K.B., Li,J., Zhu,J., Washington,M.K. and Beauchamp,R.D. (2016) Comparison of Nanostring nCounter® Data on FFPE Colon Cancer Samples and Affymetrix Microarray Data on Matched Frozen Tissues. *PLOS ONE*, 11, e0153784.
