# Supplementary material for: A random forest based biomarker discovery and power analysis framework for diagnostics research
Source: BMC Med Genomics. 2020 Nov 23;13:178. doi: 10.1186/s12920-020-00826-6 (PMC7685541; doi:10.1186/s12920-020-00826-6)
Supplement: Supplementary file 2 — Additional file 2. A user guide on PowerTools. [file 12920_2020_826_MOESM2_ESM.docx]

**Title: A Random Forest based biomarker discovery and power analysis framework for diagnostics research**

**Animesh Acharjee^1,2,3,*,#^, Joseph Larkman^1,2,*^, Yuanwei Xu^1,2^, Victor Roth Cardoso^1,2,4^, Georgios V. Gkoutos^1-6^**

^1^College of Medical and Dental Sciences, Institute of Cancer and Genomic

Sciences, Centre for Computational Biology, University of Birmingham, B15 2TT, UK

^2^Institute of Translational Medicine, University Hospitals Birmingham NHS, Foundation Trust, B15 2TT, UK

^3^NIHR Surgical Reconstruction and Microbiology Research Centre, University Hospital Birmingham, Birmingham B15 2WB, UK.

^4^MRC Health Data Research UK (HDR UK)

^5^NIHR Experimental Cancer Medicine Centre, B15 2TT, Birmingham, UK

^6^NIHR Biomedical Research Centre, University Hospital Birmingham, Birmingham, B15 2TT, UK.

*** Equal first author**

**# Corresponding Author.**

**Animesh Acharjee**

**Tel.: +44 (0)1213718135**

**E-mail: a.acharjee@bham.ac.uk**

**PowerTools user manual**

We will execute a case scenario to demonstrate PowerTools web interface [1]. The dataset was selected from Acharjee et al., 2017 [2]. This document explained in two parts: data handling and PowerTools exploration.

**Data handling**

Before starting the analysis we need to have a copy of the dataset. We load the dataset into Microsoft Excel [3]. There are many columns indicating different lipid profiles and a class for each data point indicating the type of nutrition the infant had – *Formula* for feeding exclusively with formula, *HM* indicates exclusively breastfed and *HM & formula* indicates mix-fed. The number of samples with *Formula*, *HM* and *HM & formula* nutrition are 87, 85 and 67 respectively.

We want to estimate the power when doing a classification between *Formula* and *HM*. In order to select the data to our two-class problem, we delete the *HM & formula* rows. We also remove the first column, and keep the two-group variable as the first column in the dataset. This file is then saved as a *CSV (Comma delimited) (*.csv)* format, which removes all the other sheets.

**PowerTools**

We start loading the PowerTools webpage [1] . In the *Data Upload* tab, under *Choose CSV File*, click on *Browse…* to upload the file we created. After the dataset is uploaded a red message indicating *Invalid data for regression mode* appears in the bottom left side. Under *Power Function Parameters* option *Outcome variable type*, select *Two-group Classification*. A blue message indicating *Data loaded* appears. Change the *Range of sample sizes* as appropriate. In this scenario the range selected is 30 to 300. Figure 4 shows the final setting. Click *Submit* to start the analysis.

After click to submit the options to analysis a text box on the right side appears and the progress of the analysis is shown. After the iteration through different sample sizes and the variables available, the view changes to the *Results* tab, It is possible to visualise the variables and the estimated power given the sample size. For example, lipid *TG.54.4* reaches power 1 with circa 120 samples, whilst LysoPC.20.3 with around 480 samples for using Bonferroni correction [4]. In the bottom of the page is possible to download the data for the results figure.


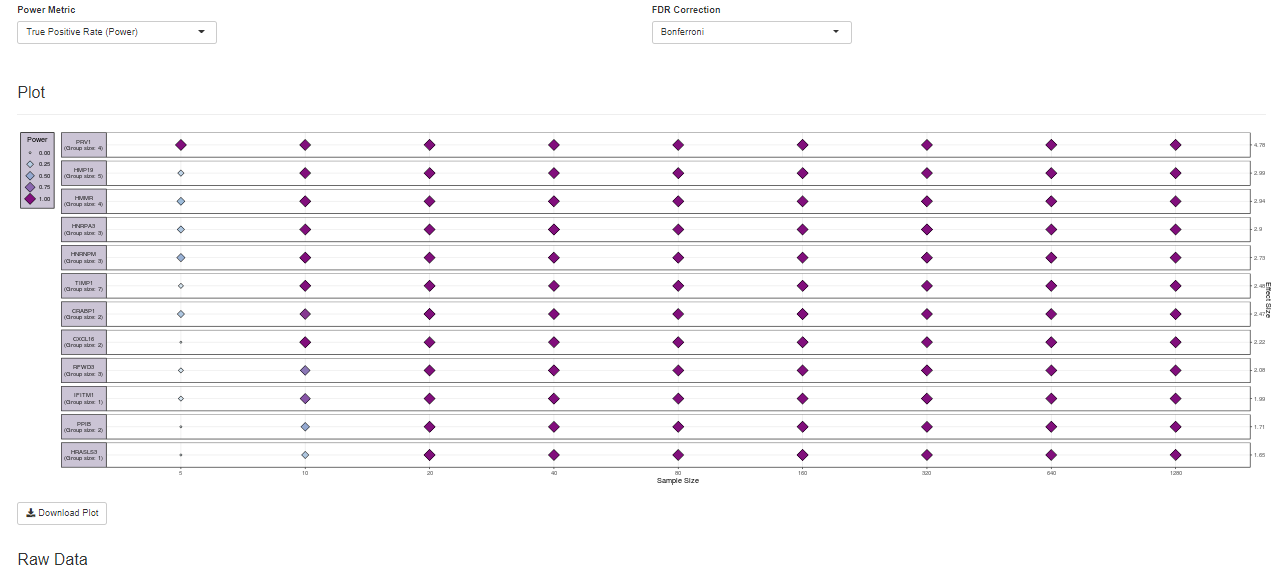


Figure : Example of the output after finishing the analysis.

**References**

1. Larkman J. PowerTools 2019 [Available from: <https://joelarkman.shinyapps.io/PowerTools/>. Accessed on 30 January 2020.

2. Acharjee A, Prentice P, Acerini C, Smith J, Hughes IA, Ong K, et al. The translation of lipid profiles to nutritional biomarkers in the study of infant metabolism. Metabolomics. 2017;13(3):25. doi:10.1007/s11306-017-1166-2.

3. Microsoft. Excel. California2016.

4. Haynes W. Bonferroni Correction. In: Dubitzky W, Wolkenhauer O, Cho K-H, Yokota H, editors. Encyclopedia of Systems Biology. New York, NY: Springer New York; 2013. p. 154-.
